# Assessing shared respiratory pathogens between domestic (*Ovis aries*) and bighorn (*Ovis canadensis*) sheep; methods for multiplex PCR, amplicon sequencing, and bioinformatics to characterize respiratory flora

**DOI:** 10.1371/journal.pone.0293062

**Published:** 2023-10-19

**Authors:** Karen A. Fox, Christopher A. W. MacGlover, Kevin A. Blecha, Mark D. Stenglein

**Affiliations:** 1 Wildlife Health Program, Colorado Parks and Wildlife, Fort Collins, Colorado, United States of America; 2 Department of Veterinary Sciences, University of Wyoming, Laramie, Wyoming, United States of America; 3 Terrestrial Branch, Colorado Parks and Wildlife, Gunnison, Colorado, United States of America; 4 Department of Microbiology, Immunology, and Pathology, College of Veterinary Medicine and Biomedical Sciences, Colorado State University, Fort Collins, Colorado, United States of America; Defense Threat Reduction Agency, UNITED STATES

## Abstract

Respiratory disease is responsible for dramatic population declines in bighorn sheep (*Ovis canadensis)*, and respiratory pathogen diagnostics contribute to the management of bighorn populations. To create a comprehensive and consistent approach to bighorn sheep respiratory diagnostics, we created a culture-independent assay to detect and strain type *Mannheimia haemolytica*, *Bibersteinia trehalosi*, *Pasteurella multocida*, and *Mycoplasma ovipneumoniae*. The assay also detects and characterizes the *Pasteurellaceae* leukotoxin A gene, and broadly assesses the bacterial composition of each sample based on 16S rRNA sequences. The assay is based on a three-step approach: 1) Multiplex PCR to amplify targets including eight loci for each bacterial species, the *Pasteurellaceae lktA* gene, and the 16S rRNA gene 2) Library preparation, barcoding, and short-read Illumina sequencing to determine the genetic sequences of each target, and 3) Bioinformatics in the form of automated software to analyze genetic sequences. The assay was designed to assess shared pathogens between domestic and bighorn sheep, but could be useful for many applications in bighorn sheep respiratory disease research and management.

## Introduction

Bighorn sheep (*Ovis canadensis*) respiratory disease and associated population declines have been documented in western North America for about a century [1]. Some bighorn populations have experienced dramatic and abrupt declines from outbreaks of epizootic pneumonia in all age classes [2–5], whereas other populations have experienced steady declines due to endemic chronic respiratory disease in the herd and annual lamb mortality [1, 6, 7]. Like respiratory diseases in domestic sheep [8] and cattle [9], bighorn sheep respiratory disease is a polymicrobial and multifactorial problem that is difficult to manage, especially in free-ranging populations [10]. Bacteria most commonly associated with respiratory disease in bighorn sheep include *Mycoplasma ovipneumoniae* [11] and bacteria from the *Pasteurellaceae* family including *Mannheimia haemolytica* [12], *Bibersteinia trehalosi* [13], and *Pasteurella multocida* [14]. These pathogens can be present in both bighorn and domestic sheep populations, and contact between bighorn and domestic sheep has been identified as a risk factor for bighorn sheep populations [15–17]. Current diagnostics to identify these bacterial pathogens in bighorn sheep include culture and PCR [18], and limited multilocus sequence typing (MLST) [19]. More robust and standardized diagnostics could be useful to provide comprehensive and consistent data for management decisions that affect both bighorn and domestic sheep stakeholders.

Multiplex PCR with amplicon sequencing is an attractive option for MLST-based respiratory diagnostics in bighorn sheep. This technology is routinely used for genomics applications and is commercially available through custom kits including TruSeq (Illumina, San Diago, CA) and Ampliseq (Thermo Fisher, Waltham, MA). These commercially available panels have been applied to MLST [20] and are often highly multiplexed, including more than 100 targets per panel [21]. Similar methods have been employed by others [22].

We had an opportunity to design improved bighorn sheep respiratory diagnostics in January, 2019 when a collared adult male Rocky Mountain bighorn sheep (*Ovis canadensis canadensis*), within a subherd of the Central San Juan bighorn herd in Colorado, USA died with severe chronic active bronchopneumonia. Although there was evidence of chronic respiratory disease in the herd prior to the death of this bighorn, the mortality was of interest due to recent documented interactions with a hobby flock of domestic (Icelandic) sheep. Because proximity to domestic sheep has been identified as a risk factor for bighorn sheep respiratory disease [15–17], there was interest in comparing the respiratory flora of the domestic sheep flock and the bighorn sheep herd to determine if pathogens had been transmitted between the two groups of animals. To address this question, and to create an assay that could be used to examine other questions related to bighorn sheep respiratory disease, we developed a culture independent MLST approach that built on existing MLST schemes and utilized multiplex PCR and next generation sequencing technology. We included 16S rRNA analysis in our assay to provide a nonbiased assessment of the bacterial flora of bighorn sheep respiratory samples. We designed automated bioinformatics to allow for rapid analysis of data from future applications of the assay.

## Materials and methods

### Reference material

We required bacterial reference sequences for assemblies and phylogenetics. Sources for reference sequences are provided in Table 1. When available, we used genomic data derived from reference (type) strains as defined by the American Type Culture Collection (ATCC) or Culture Collection University of Gothenburg (CCUG). Whole genome sequence (WGS) data was acquired for reference strains either through data provided in National Center for Biotechnology Information (NCBI) databases or, if genomic data was unavailable in NCBI, by whole genome sequencing of bacterial cultures acquired from ATCC, or by whole genome sequencing of bacterial cultures acquired courtesy of The University of Copenhagen, generously shared by Dr. H. Christensen. For whole genome sequencing of reference bacteria, we cultured bacteria on Columbia blood agar plates in 5–10% CO_2_ at 37° Celsius for 24 hours. We extracted nucleic acid following standard protocols using DNeasy blood and tissue kits (QIAgen, Valencia, CA) automated on QIAcube (QIAgen). Whole genome sequencing was performed at Wyoming State Veterinary Laboratory on an iSeq100 (Illumina) and GridION Mk1 (Oxford Nanopore Technologies, Oxford, UK) to a minimum depth of 100x. We processed sequencing data using a bioinformatics pipeline designed for whole genome hybrid de novo assembly. We identified target sequences from whole genome sequence data using Geneious (v 2022.2.2) annotations and tracks menu, and the Geneious test with saved primers function. We concatenated target gene sequences from each species to create MLST reference sequences.

**Table 1:** Reference strains used for reference-based assemblies and phylogenetic comparisons for multilocus sequence typing of bighorn sheep respiratory pathogens.

Organism	Reference Strain Source	CCUG^d^ ID	NCTC^e^ ID	NCBI^f^ ID	WGS^g^	Host
*Bibersteinia trehalosi*	ATCC^c^ 29703	n/a	NCTC 10370	Unavailable	Yes	Ovine
*Mannheima haemolytica*, GT1^a^	ATCC 33396	n/a	NCTC 9380	GCF_900452985.1	No	Ovine
*Mannheima haemolytica*, GT2^b^	USMARC 191	n/a	n/a	CP023044	No	Bovine
*Mannheimia varigena*	Courtesy of H. Christensen	38462	n/a	Unavailable	Yes	Bovine
*Mannheimia glucosida*	Courtesy of H. Christensen	38457	n/a	Unavailable	Yes	Bovine
*Mannheimia ruminalis*	Courtesy of H. Christensen	38470	n/a	Unavailable	Yes	Bovine
*Mycoplasma ovipneumoniae*	ATCC 29419	n/a	NCTC 10151	LR215028.1	No	Ovine
*Pasteurella multocida*	ATCC 43137	n/a	NCTC 10322	NZ_LT906458.1	No	Porcine

^a, b^ GT1 and GT2 = *Mannheimia haemolytica genotypes* 1 and 2, as defined by Clawson et al. [23]. The genotype 1 strain is the *M*. *haemolytica* type strain.

^c^ATCC = American Type Culture Collection

^d^CCUG = Culture Collection University of Gothenburg

^e^NCTC = National Collection of Type Cultures

^f^NCBI = National Center for Biotechnology Information

^g^WGS = Whole genome sequencing was performed to provide data for this project. Relevant gene sequences have been deposited in GenBank (Accessions OQ383448-OQ383479)

### Samples from suspected contact event between domestic and bighorn sheep

We collected antemortem and postmortem samples from animals involved in a suspected contact event between domestic and bighorn sheep [24]. Following suspected contact, a collared adult male bighorn sheep was found dead, with evidence of chronic active bronchopneumonia observed at necropsy. Tissues from the deceased bighorn sheep ram were collected within 48 hours postmortem. The in-contact domestic sheep herd was a hobby flock of three Icelandic sheep including one adult male and two adult females. The adult male Icelandic sheep was culled and tissues were collected for culture and DNA extraction. Incidentally, although this animal was determined to be apparently healthy by the owner’s veterinarian at the time of euthanasia, we identified chronic upper respiratory disease in this individual at necropsy, with abundant mucinous exudates present within the paranasal sinuses. The two remaining adult female Icelandic sheep from the in-contact hobby flock were sampled antemortem, with nasal and oropharyngeal swabs placed in Amies transport media and shipped overnight to the Wyoming Game and Fish Department bacteriology lab. Within 48 hours, the swabs were used to inoculate CBA agar plates and modified tryptone soya broth for *Pasteurellaceae* and *Mycoplasma* growth, respectively [18, 25]. *Mycoplasma* broth and *Pasteurellaceae* culture plates were incubated at 37°C and 10% CO_2_ for 36–48 hours. Bacterial colonies were identified phenotypically, and by conventional PCR of culture plate washes [18]. The culture plates were washed with 13 ml PBS, vortexed, and 200 μl was aliquotted for nucleic acid extraction. If growth was observed in the *Mycoplasma* broth, 1 ml was aliquoted, and centrifuged for removal of supernatant. The bacterial pellet was resuspended in 200 μl of PBS, and vortexed, for nucleic acid extraction. Extraction was automated on QIAcube using DNeasy Blood and Tissue standard kit protocol, according to manufacture specifications (QIAgen).

**Fig 1:**
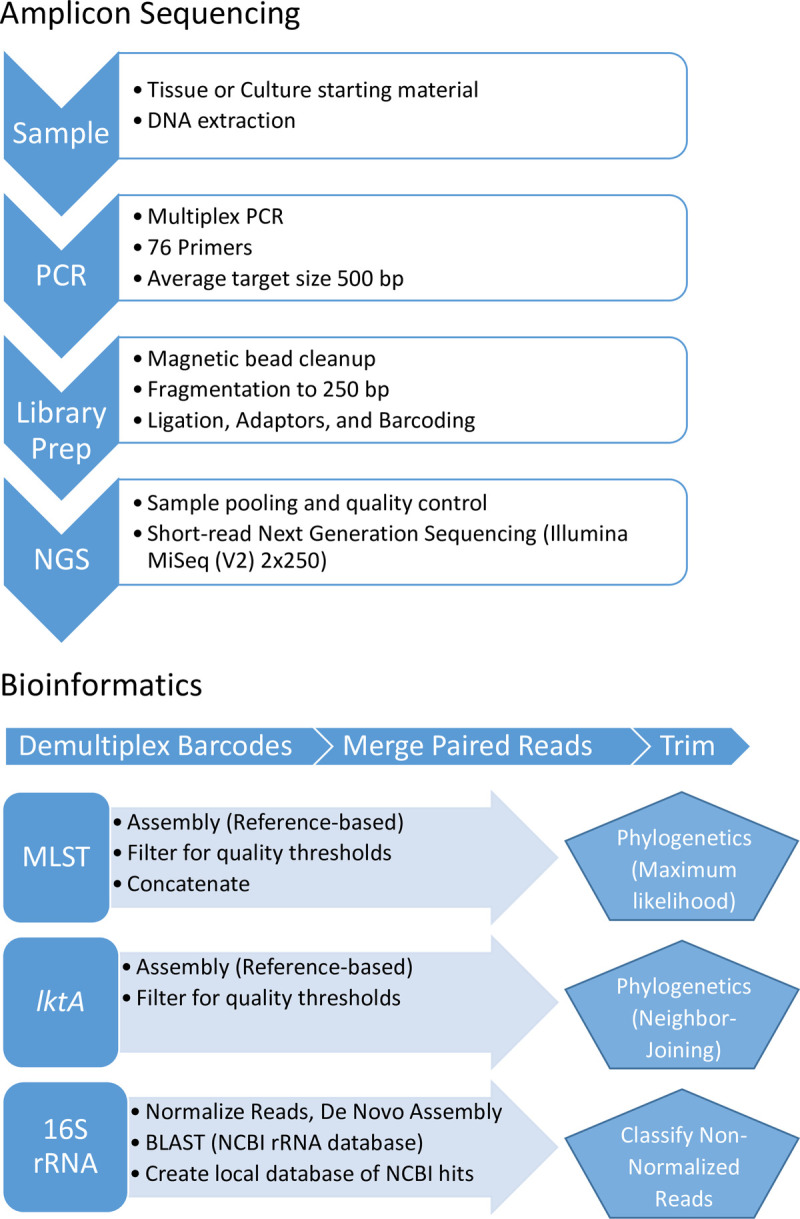
Graphical representation of assay workflow. The starting sample of fresh tissue, or bacterial culture sample is processed with DNA extraction followed by multiplex PCR, library preparation, and amplicon sequencing. Bioinformatics are automated through the Geneious software platform with necessary plugins. Workflows for MLST and *lktA* use a reference-based assembly algorithm while the 16S rRNA workflow uses a de novo assembly approach. Final results are presented as phylogenetic trees (MLST, *lktA*) or classified sequence data (16S rRNA).

Samples used for the comparison of respiratory flora between the domestic and bighorn sheep groups included: Lung, maxillary sinus lining, and tonsil tissues collected approximately 48 hours postmortem from the deceased adult male bighorn sheep (BHS 18 181) that died in January, 2018 with pneumonia; paranasal sinus lining from an adult male bighorn sheep (BHS 18 235) that belonged to the same wild subherd and died after becoming entangled in a fence in May of 2017; lung tissue from an adult male bighorn sheep (BHS 18 635) that belonged to the same wild subherd and died from malnutrition due to a gingival sarcoma in April, 2018; lung, maxillary sinus lining, and maxillary sinus exudates from the adult male Icelandic sheep (DS 18 219) that was culled from the hobby flock February 7, 2018; bacterial cultures from tissues of the adult male Icelandic sheep that was culled; and bacterial cultures from nasal and tonsil swabs taken February 7, 2018 from the two live adult female Icelandic sheep (DS 18 220, DS 18 221) that remained in the hobby flock. All tissues, culture plate washes, and culture broth were preserved at -80°C prior to DNA extraction. For DNA extractions, we used DNeasy blood and tissue kits (QIAgen) according to manufacturer instructions. A graphical representation of our workflow is provided in Fig 1 (Fig 1).

### Primer design and multiplex PCR

We used a multiplex PCR design with 38 primer pairs targeting genes of four bacterial species (*M*. *haemolytica*, *B*. *trehalosi*, *P*. *multocida*, and *M*. *ovipneumoniae*), the *Pasteurellaceae* leukotoxin A gene (*lktA*), and the 16S rRNA gene. In general, primers were selected to amplify targets approximately 500 base pairs (bp) in length. Few targets were greater than 900 bp in length, and we included a proportionately higher concentration of primers for these targets in the final primer mix. To increase sensitivity for some loci, we included a second set of primers external to the targeted sequence (S1 Table). We used published MLST schemes (pubMLST [26]) for *M*. *haemolytica* and *P*. *multocida* as a basis for our MLST approach to the *Pasteurellaceae* bacteria, with modifications based on preliminary results using these primers, and other literature referencing housekeeping genes in *Pasteurellaceae* bacteria [27]. Primers previously used for *M*. *ovipneumoniae* MLST by others [19, 28, 29] were included in our MLST scheme for backward comparisons, although this approach targets only three genes (gyrB, rpoB, and 16S rRNA) as well as an intergenic spacer region [19]. We expanded this MLST approach to provide a total of eight targets. Final MLST loci are provided in Table 2.

**Table 2:** Multilocus sequence typing schemes for bighorn sheep respiratory pathogens targeted by multiplex PCR.

Organism	MLST loci
*Bibersteinia trehalosi*	*adk*, *atpD*, *deoD*, *gapDH*, *infB*, *mdh*, *rpoB*, *zwf*
*Mannheimia haemolytica*	*adk*, *aroE*, *deoD*, *gapDH*, *gnd*, *mdh*, *rpoB*, *zwf*
*Pasteurella multocida*	*adk*, *deoD*, *gapDH*, *gdhA*, *infB*, *mdh*, *pgi*, *rpoB*
*Mycoplasma ovipneumoniae*	*gyrB*, IGS, *16S rRNA*, *rpoB*, *adk*, *atpA*, *gltX*, *tpiA*

The primer mix was prepared with 1X TE Buffer. PCR reactions were prepared with a Multiplex PCR kit (QIAgen), with a final concentration of 1X Master mix, 1X Q solution, primer concentrations as described in S1 Table, and 25–500 ng of template DNA to a final volume of 12.5 μl. PCR cycling conditions included an initial denaturation step at 95˚C for 15 minutes, followed by 40 cycles of 94˚C for 30 seconds, 59˚C for 3 minutes, and 72˚C for 90 seconds, with a final extension step of 68˚C for 15 minutes. PCR products were visualized by electrophoresis on a 1.5% agarose gel. For PCR cleanup we used AMPure XP (Beckman Coulter, Brea, CA) magnetic beads, selecting for DNA fragments greater than 300 bp. For each sample, we used a ratio of 6 μl of beads and 10 μl of PCR product, with incubations and washes according to manufacturer instructions. We transferred 18 μl of final DNA solution to a new tube and measured DNA concentration with a NanoDrop Lite spectrophotometer (Thermo Fisher).

### Library preparation and short read sequencing

For library preparation we used a NEBNext Ultra FS DNA Library Prep Kit for Illumina (New England Biolabs, Ipswitch, MA) and NEBNext Multiplex Oligos for Illumina (New England Biolabs) following manufacturer instructions including optional overnight freezing steps. For each sample, DNA amplified by multiplex PCR was fragmented to a target length of 250 bp using 200 ng of DNA, 7 μl of Ultra II FS buffer, 2 μl of Ultra II FS Enzyme Mix, and 1X TE buffer to a final volume of 35 μl. The reaction was incubated in a PTC-100 Peltier thermocycler (MJ Research, Inc., Deltona, FL) with lid set at 75˚C. Incubation was at 37˚C for 4 minutes followed by 65˚C for 30 minutes and held at 4˚C. Fragmented DNA was transferred to a -20˚C freezer overnight. We adaptor-ligated DNA fragments using NEBNext Ultra II Ligation Master Mix, NEBNext Ligation Enhancer, NEBNext Adaptor, and USER enzyme according to manufacturer instructions. Ligations were transferred to a -20˚C freezer overnight. We selected for ligations with inserts of 250 bp using AMPure (Beckman Coulter) beads according to manufacturer (New England Biolabs) instructions. We transferred 15 μl of each size-selected ligation reaction mixture to a new tube and froze overnight at -20˚C. Barcoding was performed using Dual Index Primer Pairs according to manufacturer’s instructions. For each sample, 15 μl of ligation reaction mixture was combined with 25 μl of Q5 Master Mix and 10 μl unique dual index primers to a final volume of 50 μl. Amplification was performed with PCR conducted at one cycle of 98 ˚C for 30 seconds, followed by 4 cycles of 98˚C for 10 seconds and 65˚C for 75 seconds, a final extension at 65˚C for 5 minutes, and held at 4˚C. Final PCR cleanup was performed with AMPure (Beckman Coulter) magnetic beads according to manufacturer (New England Biolabs) instructions. We transferred 30 μl of each final library to a new tube and froze at -20˚C.

Final libraries were quantified using an Invitrogen Qubit dsDNA HS Assay kit (Invitrogen, Waltham, MA) and a Qubit fluorometer (Thermo Fisher). Samples were pooled to include approximately equivalent concentrations, with a final pooled library concentration of approximately 2.6 ng/μL. Libraries were sequenced using an Illumina MiSeq (V2) 2x250 kit. Results were demultiplexed by barcodes. We imported and merged paired reads through the Geneious FASTQ import function, with options for paired ends (inward pointing) and insert size 250 bp. We trimmed adapter sequences, low quality reads, and short reads using the Geneious BBDuk plugin (v 38.84) with parameters provided in S2 Table.

### Geneious workflows

We created workflows for bioinformatics using the Geneious (v 2022.2.2) software platform and available plugins. Full Geneious workflows and reference sequences are provided through the Github Repository [30].

#### MLST workflow

To identify target sequences for MLST analysis, we used a reference-based assembly approach [31]. A reference-based assembly approach uses target reference sequences to guide identification, alignment, and assembly of DNA fragments into contiguous representations (contigs) of DNA amplicons. In our application, assembled (consensus) sequences used for downstream analyses represented the predominant sequence/strain that was amplified for each target. Each MLST target locus was evaluated independently, and a locus was only included in the MLST analysis if the consensus sequence met quality thresholds. Loci were then concatenated to create full MLST sequences for phylogenetics. If some loci did not produce an adequate consensus sequence, the resulting concatenated sequences contained missing data. Only assemblies with at least four (three for *M*. *varigena*) of the eight target loci assembled were used for phylogenetics.

We performed reference-based assemblies (Geneious Bowtie2 Plugin (version 7.2.1) with parameters provided in S3 Table. For each sample library, we assembled reads to all reference sequences (n = 7; *M*. *haemolytica*, *M*. *ruminalis*, *M*. *glucosida*, *M*. *varigena*, *B*. *trehalosi*, *P*. *multocida*, and *M*. *ovipneumoniae*) with assembly to the best match only. We filtered the resulting contigs by corresponding reference sequence, dissolved the filtered contigs, and re-assembled the reads (S4 Table) to the individual MLST loci of the corresponding reference sequence with primers trimmed. The consensus sequences (S5 Table) for each target were concatenated by assembly to concatenated reference sequences (S6 Table).

#### *Pasteurellaceae lktA* workflow

Similar to the MLST workflow, a reference-based assembly was used for *lktA* analysis, with quality thresholds. Reference sequences for ten *lktA* alleles representing *M*. *haemolytica*, *M*. *glucosida*, and *B*. *trehalosi* were obtained from Davies et al. [32] and are provided in Table 3. Although each sequence represents a unique *lktA* allele, several sequences shared >98% homology based on the internal sequence targeted with primers for this analysis (see *lktA* allele matrix, S7 Table). To allow optimal mapping, highly similar sequences were represented by a single sequence. Additionally, representative *lktA* sequences from *M*. *varigena* and *M*. *ruminalis* were also included. A total of seven *lktA* reference sequences were used for reference-based assemblies and phylogenetics (Table 3). Assembly by mapping to reference *lktA* alleles was performed using the Bowtie2 plugin (version 7.2.1) for Geneious. For each sample, reads were assembled to the seven reference alleles with parameters provided in S8 Table. For each reference sequence (per library), saved contigs were dissolved into original reads and re-assembled to reference sequences with primers trimmed, using the Geneious Map to Reference feature, trimming to reference sequence (S9 Table) and filtering for no ambiguities.

**Table 3:** *Pasteurellaceae* leukotoxin A sequences considered as reference sequences for reference-based assemblies and phylogenetics.

Lkt allele^a^	GenBank Accession	Associated Species	Included as Reference	Notes
A1.1	AF314503	*M*. *haemolytica*	No	Not included as reference, 98.01% similar to lktA4.1 (*M*. *glucosida*) in this region
A2.1	AF314511	*M*. *haemolytica*	Yes	
A3	AF314512	*M*. *haemolytica*	Yes	
A4.1	AF314517	*M*. *glucosida*	Yes	
A5.1	AF314523	*B*. *trehalosi*	Yes	
A6	AF314510	*M*. *haemolytica*	No	Not included as reference, 99.78% similar to lktA5.1 (*B*. *trehalosi*) in this region
A7	AF314509	*M*. *haemolytica*	No	Not included as reference, 98.57% similar to lktA8.1 and lktA9 (*M*. *haemolytica*) in this region
A8.1	AF314515	*M*. *haemolytica*	Yes	
A9	AF314508	*M*. *haemolytica*	No	Not included as reference, 100% similar to lktA8.1 (*M*. *haemolytica*) in this region
A10.1	AF314514	*M*. *haemolytica*	No	Not included as reference, 100% similar to lktA1.1 (*M*. *haemolytica*) in this region
n/a	n/a	*M*. *varigena*	Yes	*lktA* sequence identified in WGS assembly performed for this project
n/a	AY425280.2	*M*. *ruminalis*	Yes	

^a^Alleles as described by Davies et al. (2001).

#### 16S rRNA workflow

The 16S rRNA primers used in this study targeted the V1-V9 region, with nested primers targeting the V3-V4 region (see S1 Table). Given the lack of a degenerate reference sequence for the 16S rRNA gene, a reference-based assembly for 16S rRNA analysis was not feasible. To identify and classify 16S rRNA reads, for each library we first error corrected and normalized the reads (BBNorm v 38.84, S10 Table) to reduce computational burden in high-depth regions, and then completed a de novo assembly of each library (Geneious v 2022.2.2, S11 Table). The de novo assembly was slower than our reference-based assemblies, but allowed DNA amplicons to be assembled without comparison against a known target sequence. The resulting contigs from each sample were then compared against the 16S rRNA NCBI database [33]. Unique hits were saved to a local database, against which reads could then be classified to determine relative prevalence of each 16S sequence among each sample’s 16S rRNA amplicons. In constructing our local database, we also wanted to assure that the reference strains used for the MLST assays were represented in the local database. We determined the 16S rRNA gene sequences of our reference strains using WGS data from NCBI or in-house WGS efforts as described in Table 1. We compared each 16S rRNA reference gene against the NCBI 16S rRNA database [33] and found all of our reference strains to be represented (>99.5% homology across V1-V9), except for the *B*. *trehalosi* type strain for which the closest NCBI 16S rRNA hit had only 96.4% homology across the gene (V1-V9). We therefore added the reference *B*. *trehalosi* 16S rRNA gene to our local database and proceeded with read classification. The full set of library reads (prior to normalization) for each library was then classified against our local database using the Geneious Classify Sequences Tool (S12 Table).

### Tree building

MLST sequences generated for bacterial strains were aligned (MAFFT alignment) with type strain reference sequences as outgroups to root the trees. Trees were generated using a maximum likelihood algorithm (S13 Table). Data generated from each library were included in up to four maximum likelihood phlylogenetic trees–one each for *Mannheimia* sequences, *B*. *trehalosi* sequences, *P*. *multocida* sequences, and *M*. *ovipneumoniae* sequences. For the *lktA* workflow, due to horizontal gene transfer of the *lktA* gene, a neighbor-joining tree (S14 Table) was used instead of a maximum likelihood tree. No outgroup was used for the neighbor-joining tree, but all sequences listed in Table 3 were included in the alignment and tree.

### Validation approach

To validate the MLST and *lktA* workflows, we analyzed libraries generated from type strain bacteria and compared the results against the reference sequences for those type strains. To assure that mixed populations of similar bacteria were accurately identified, we created artificial datasets by mixing reads from type strain libraries. We performed this exercise using mixtures of reads from *M*. *haemolytica* with *M*. *glucosida*, *M*. *haemolytica* with *M*. *ruminalis*, and *M*. *haemolytica* with *B*. *trehalosi*. Library reads were randomly subsampled using the Geneious Randomly Subsample Sequences workflow and combined at ratios of 1:99, 10:90, 25:75, 50:50, 75:25, 90:10, and 99:1 with a total of 500,000 reads for each combination.

## Results

### MLST results

**Fig 2:**
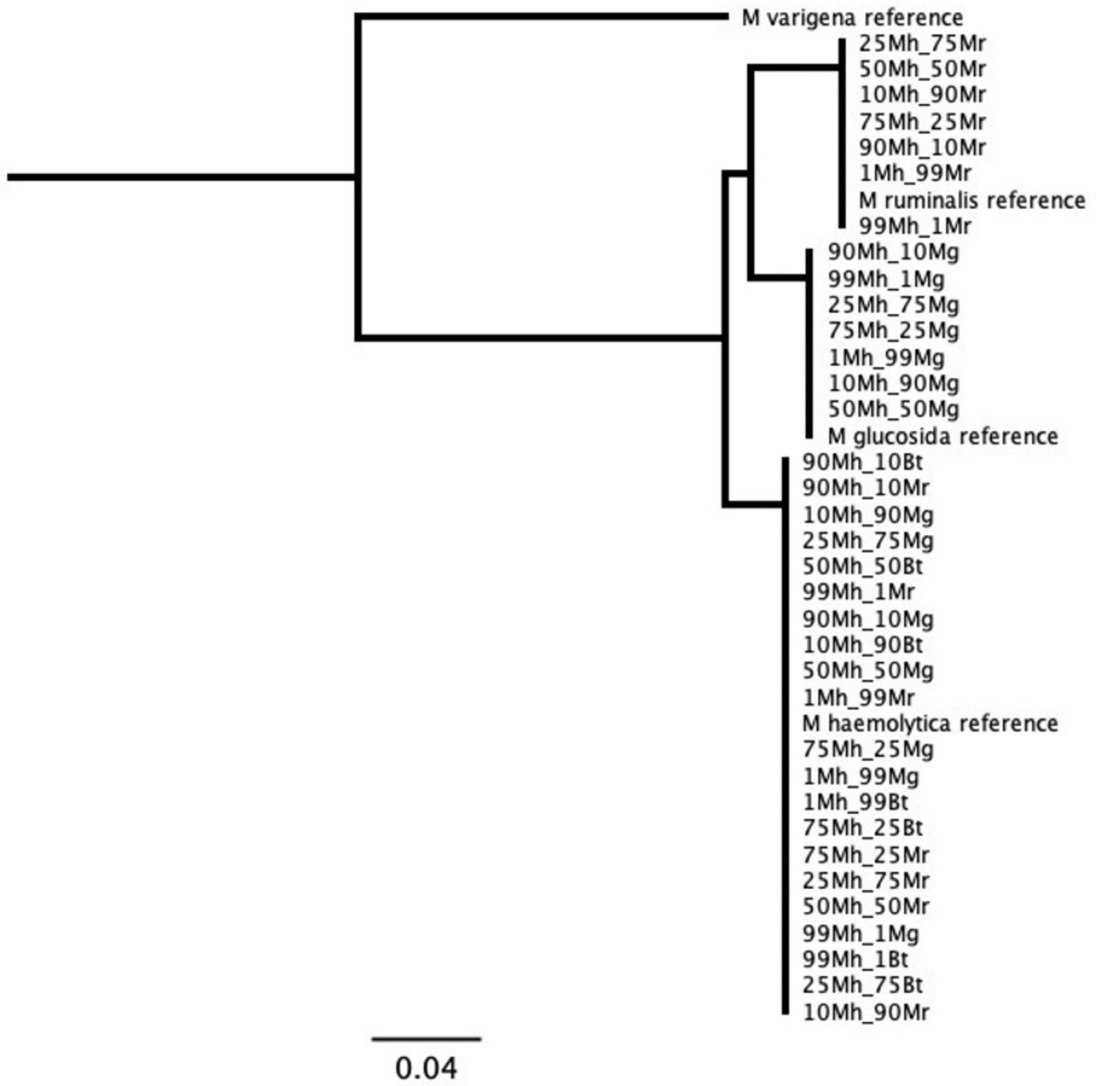
MLST results for *Mannheimia* sequences using mixed reads from closely-related species. Numbers in data labels represent proportion of reads from each of two mixed type strain libraries. Mh = *Mannheimia haemolytica*; Mr = *Mannheimia ruminalis*; Mg = *Mannheimia glucosida*; Bt = *Bibersteinia trehalosi*. Tree produced with maximum likelihood algorithm and *Mannheimia varigena* reference as root.

**Fig 3:**
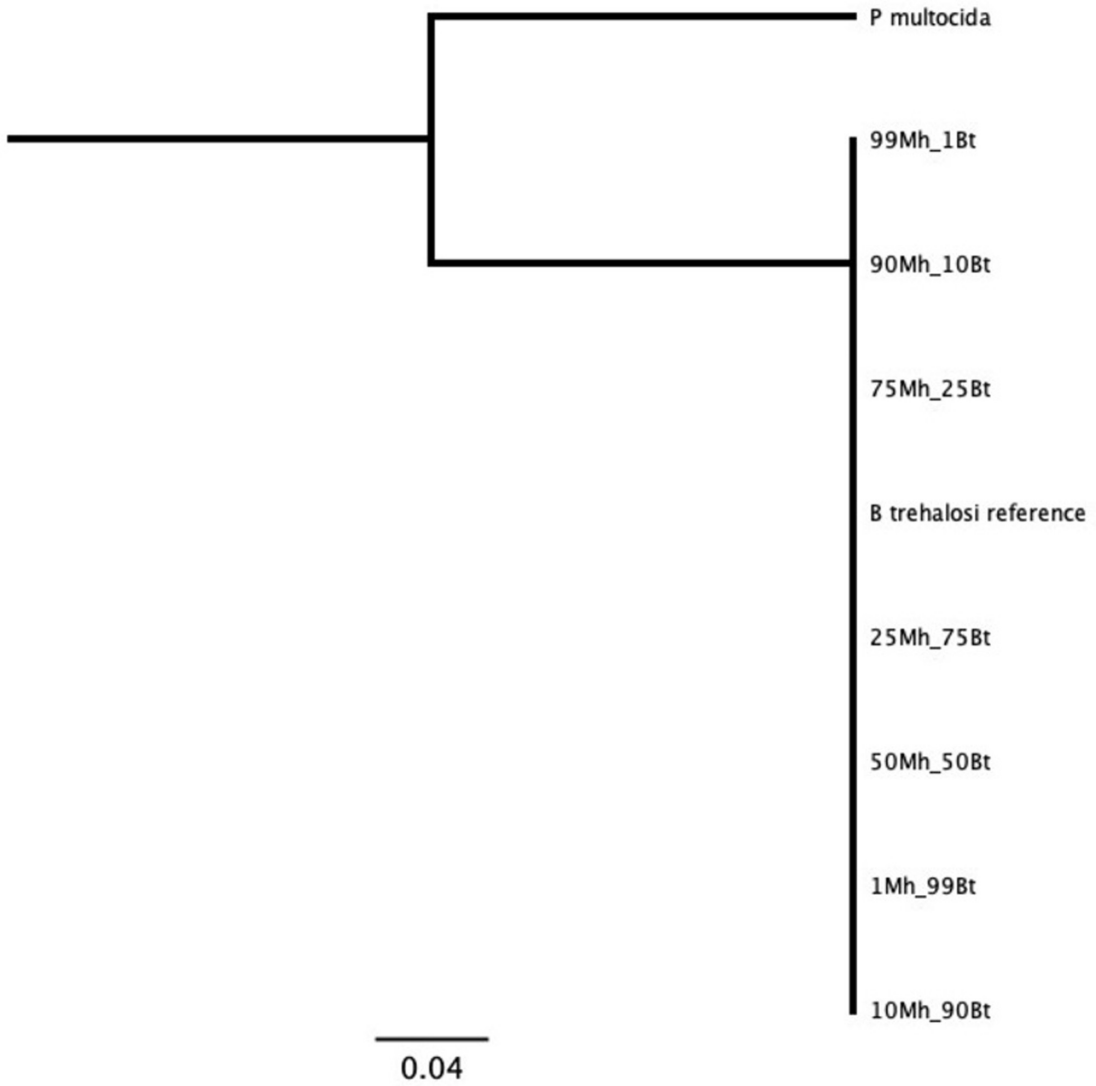
MLST results for *Bibersteinia trehalosi* sequences using mixed reads from *Mannheimia haemolytica* and *B*. *trehalosi* type strain libraries. Numbers in data labels represent proportion of reads from each of the two mixed libraries. Mh = *Mannheimia haemolytica*; Bt = *Bibersteinia trehalosi*. Tree produced with maximum likelihood algorithm and *Pasteurella multocida* reference as root.

MLST results from libraries generated from type strain bacteria matched the expected reference sequences for those type strains (S1 Fig). As expected, not all *M*. *ruminalis*, *M*. *glucosida*, and *M*. *varigena* genes were detected by the multiplex assay, as primers were not designed specifically for these species, and any amplification was due to nontarget amplification by the *M*. *haemolytica* primers. For each mixture of library reads from two closely-related reference bacteria type strains, the workflow detected two unique sequences at all levels of mixtures, with 100% similarity within each unique sequence. Minimal loss of sensitivity occurred at 10% and 1% of the mixture for *B*. *trehalosi* and *M*. *glucosida* with dropout of one or two genes (Figs 2 and 3 and S2).

### *Pasteurellaceae lkt A* phylogeny

**Fig 4:**
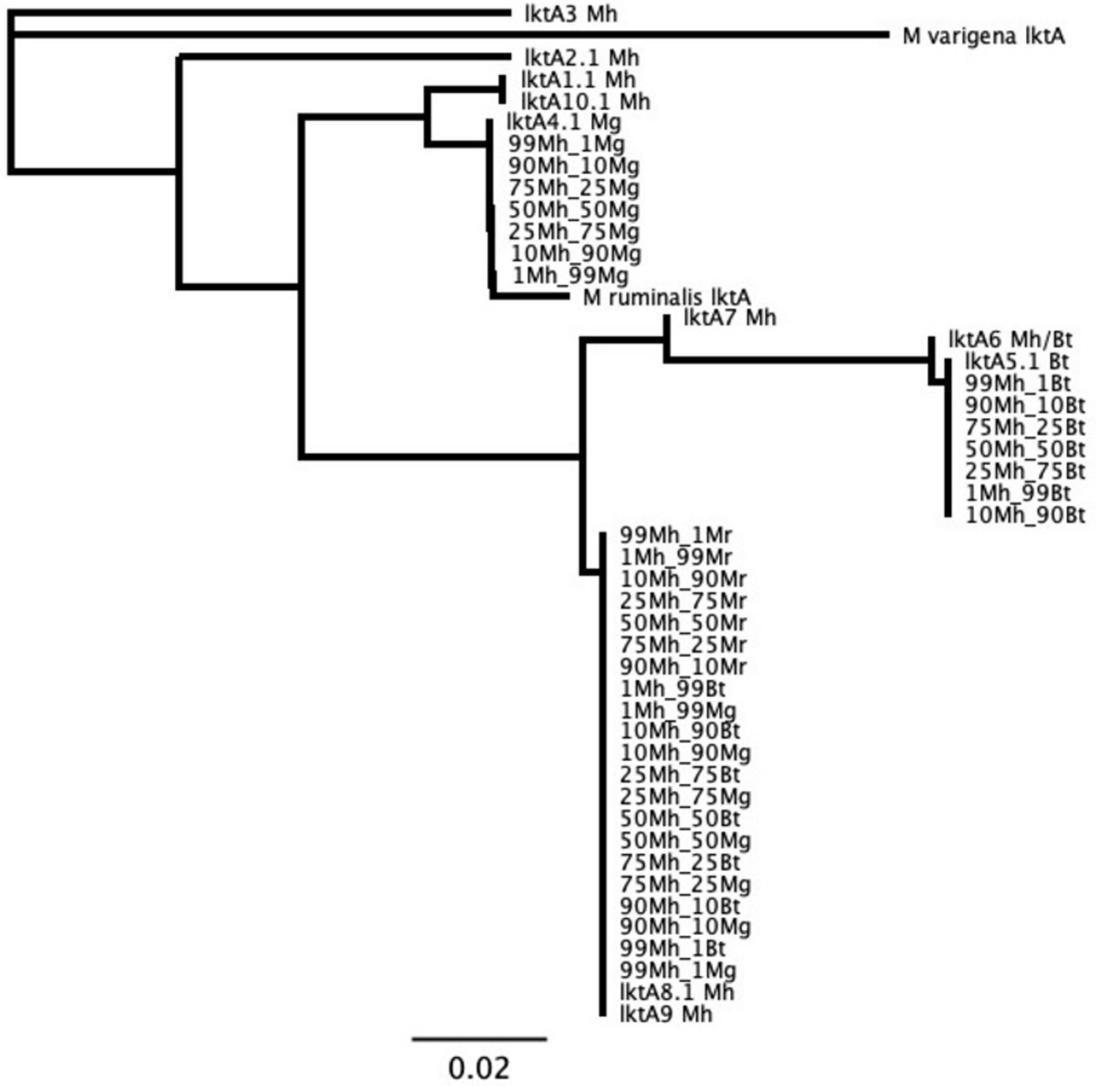
Phylogenetic tree of *Pasteurellaceae* leukotoxin A using mixed reads of closely-related type strain bacteria. Numbers represent proportion of reads from each of two mixed type strain libraries. Mh = *Mannheimia haemolytica*; Mr = *Mannheimia ruminalis*; Mg = *Mannheimia glucosida*; Bt = *Bibersteinia trehalosi*. Tree produced with neighbor-joining algorithm and no outgroup.

Results for *lktA* sequences generated from libraries of type strain bacteria matched the expected reference sequences for those type strains. For *M*. *varigena*, no *lktA* sequences were identified by the *lktA* analysis. This is likely due to primer mismatching (S3 Fig), as the *lktA* gene was found in the *M*. *varigena* type-strain WGS data. No *lktA* sequences were identified for the *M*. *ruminalis* type strain, consistent with the lack of *lktA* sequences in the WGS data generated from this sample. For each combination of library reads from two type strain bacteria with different *lktA* genes, the workflow detected two unique *lktA* sequences at all levels of mixtures, with 100% similarity within each of the two unique sequences (Fig 4). Again, no *lktA* sequences were identified as *M*. *varigena* or *M*. *ruminalis*.

### 16S rRNA analysis

For most type strain bacteria libraries, coverage of the 16S rRNA gene from V1-V4 was generally high, with lower coverage from V4-V9. For *M*. *ovipneumoniae*, coverage was limited to the V1-V3 region of the 16S rRNA gene, consistent with the inclusion of 16S primers in the MLST scheme, and favoring amplification of a smaller product (S4 Fig). 16S rRNA sequence classification results from type strain bacteria were accurate, with less than 0.1% of classified reads classified as the incorrect genus (Table 4). For most of the type strain bacteria, greater than 98% of classified reads were classified to the correct genus. For *B*. *trehalosi* and *M*. *ruminalis*, approximately 13% of the classified reads matched equally well to both reference sequences, and therefore were classified at the next highest classification, family *Pasteurellaceae* (Table 4).

**Table 4:** Results from 16S rRNA analysis for type strain bacteria.

Library	Total reads	Reads classified	% Reads classified	Reads correctly classified to genus	Reads correctly classified > genus^a^	Reads classified to incorrect genus	% Correct genus	% Incorrect genus	% Correct > genus
*M*. *haemolytica*	486,406	5,059	1.04%	5,004	54	1	98.91%	0.02%	99.98%
*P*. *multocida*	571,710	46,670	8.16%	45,794	865	11	98.12%	0.02%	99.98%
*B*. *trehalosi*	586,744	18,119	3.09%	15,642	2,460	17	86.33%	0.09%	99.91%
*M*. *ovipneumoniae*	510,340	73,234	14.35%	72,882	349	3	99.52%	0.00%	100.00%
*M*. *ruminalis*	504,300	44,031	8.73%	38,670	5,313	48	87.82%	0.11%	99.89%
*M*. *varigena*	566,164	133,424	23.57%	131,313	1,998	113	98.42%	0.08%	99.92%
*M*. *glucosida*	651,752	21,139	3.24%	20,854	266	19	98.65%	0.09%	99.91%

^a^ > genus represents classification to a taxonomic level higher than genus, for example classification may have only been possible to the level of family.

### Results for samples from suspected contact event between domestic and bighorn sheep

**Fig 5:**
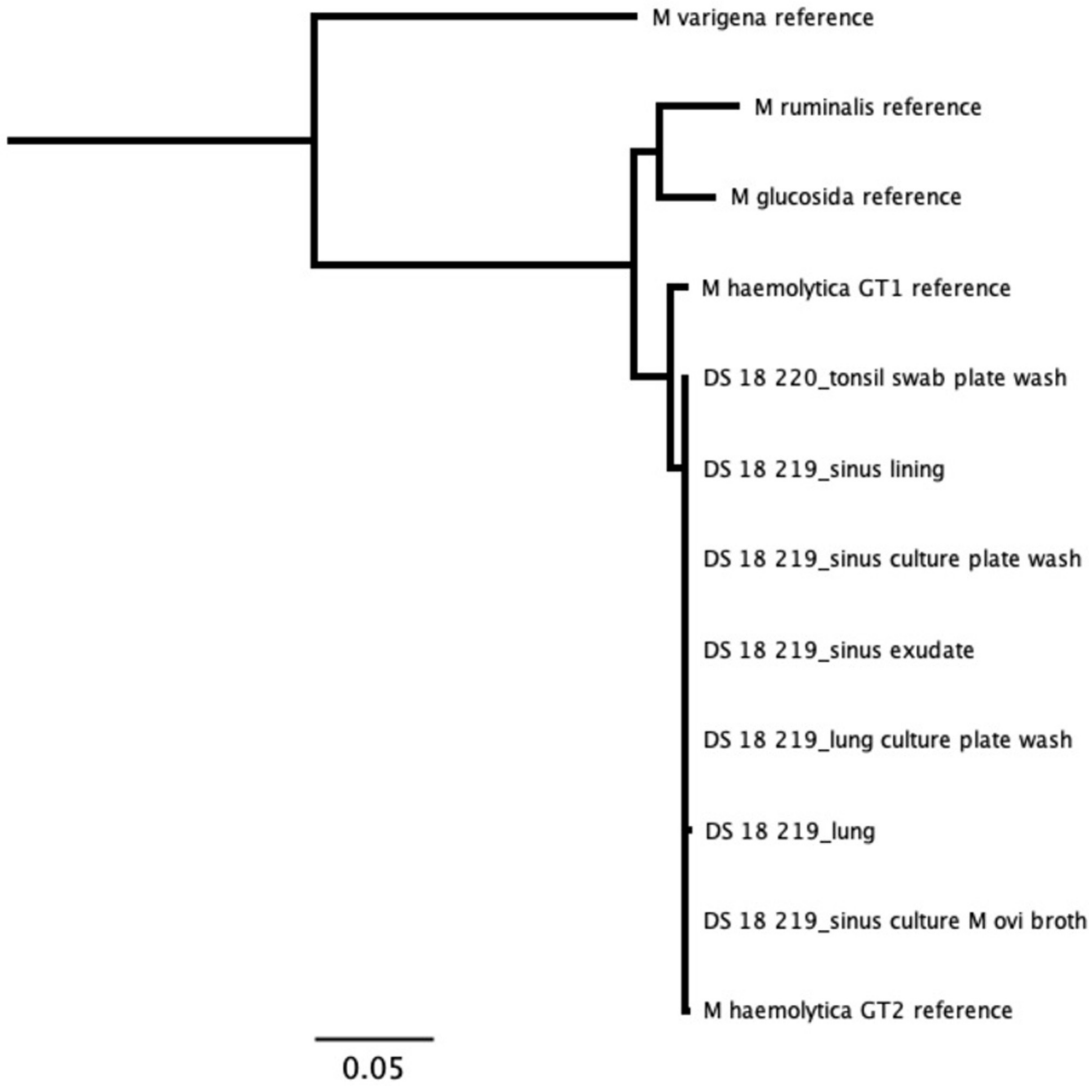
*Mannheimia* MLST results for samples from in-contact bighorn and domestic sheep. *Mannheimia* sequences were only identified in domestic sheep samples. All sequences tightly clustered with the *M*. *haemolytica* genotype 2 reference sequence. DS = domestic sheep; Tree produced with maximum likelihood algorithm and *M*. *varigena* reference as root.

**Fig 6:**
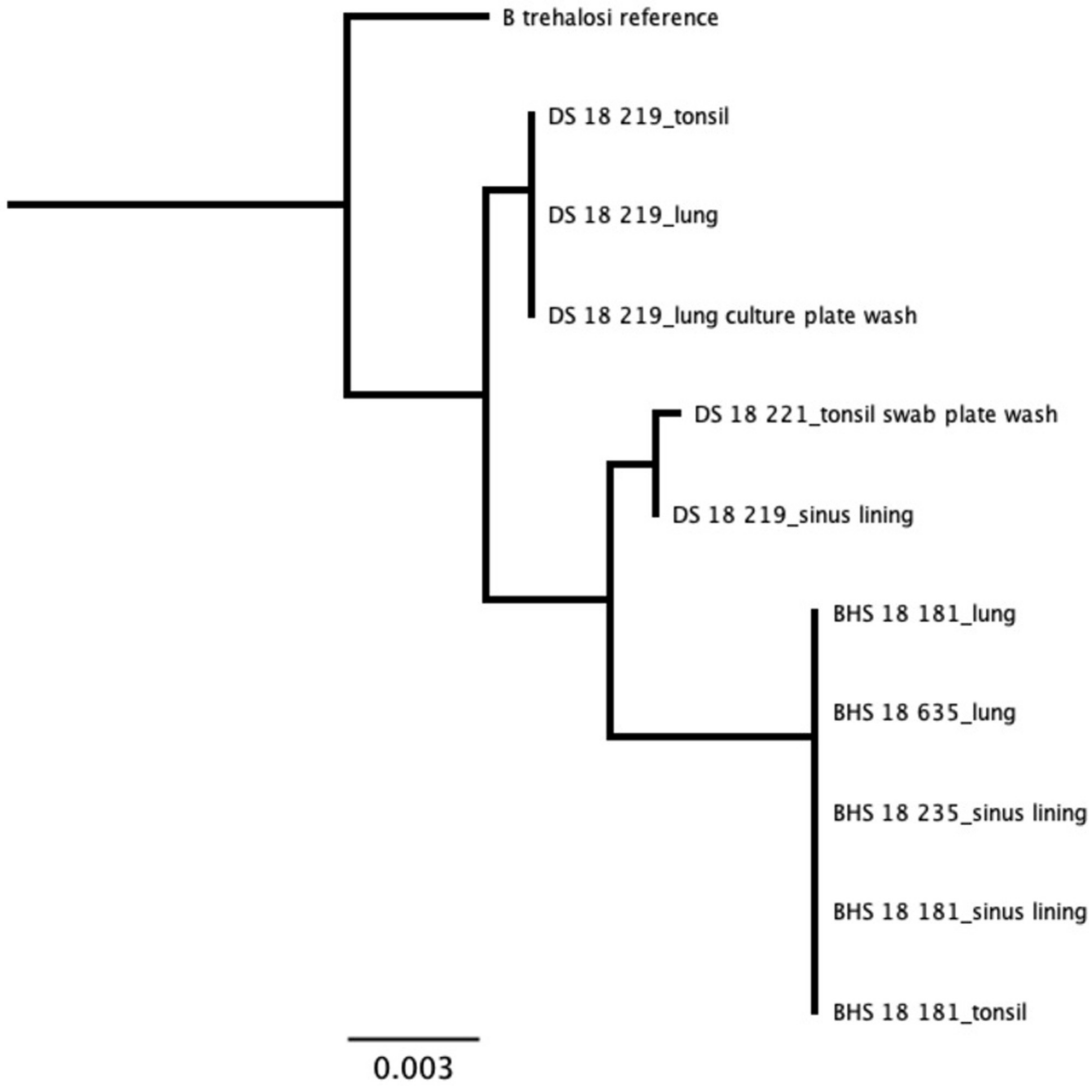
*Bibersteinia trehalosi* MLST results for samples from in-contact bighorn and domestic sheep. One unique strain was identified from the bighorn sheep samples, and two additional unique strains were identified from the domestic sheep samples. DS = domestic sheep; BHS = bighorn sheep. Tree produced with maximum likelihood algorithm and *B*. *trehalosi* reference as root.

**Fig 7:**
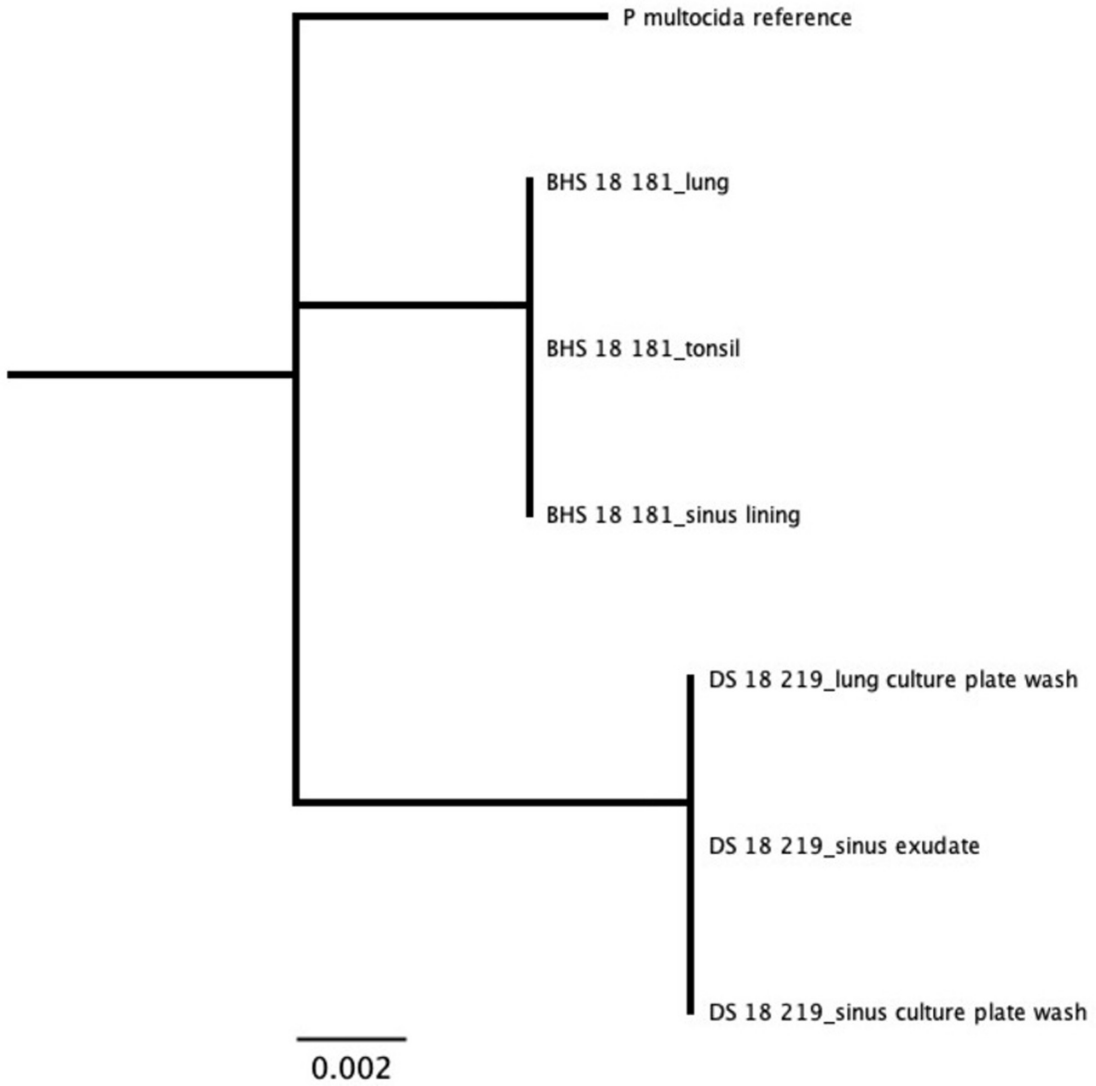
*Pasteurella multocida* MLST resultsfor samples from in-contact bighorn and domestic sheep. Sequences were identified in only two animals, and two unique strains were identified, one for each individual. DS = domestic sheep; BHS = bighorn sheep. Tree produced with maximum likelihood algorithm and *Pasteurella multocida* reference as root.

**Fig 8:**
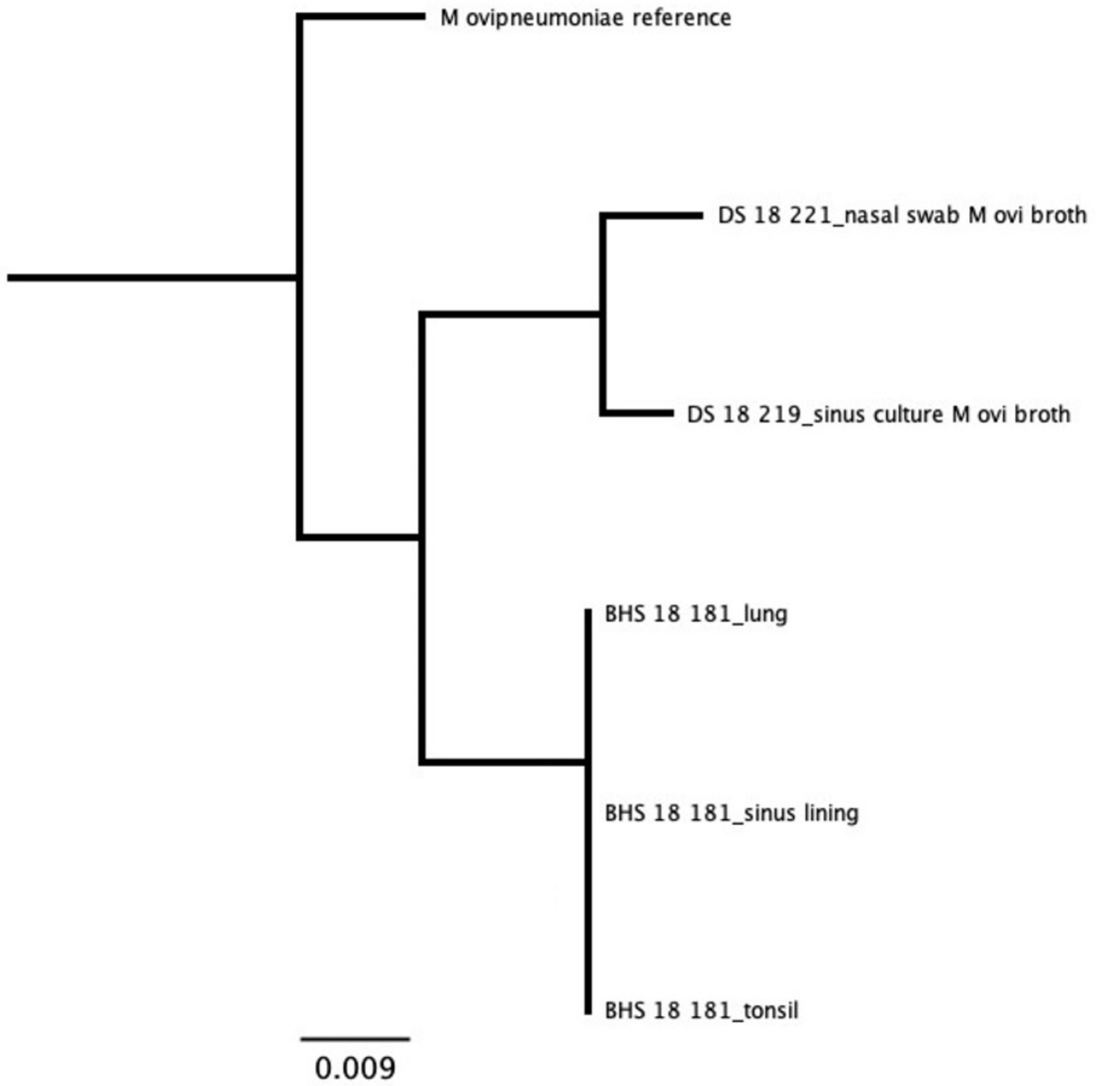
*Mycoplasma ovipneumoniae* results for samples from in-contact bighorn and domestic sheep. Sequences were identified in one bighorn sheep and two domestic sheep. Two unique strains were identified, one from the domestic sheep and one from the bighorn sheep. DS = domestic sheep; BHS = bighorn sheep. Tree produced with maximum likelihood algorithm and *M*. *ovipneumoniae* reference as root.

**Fig 9:**
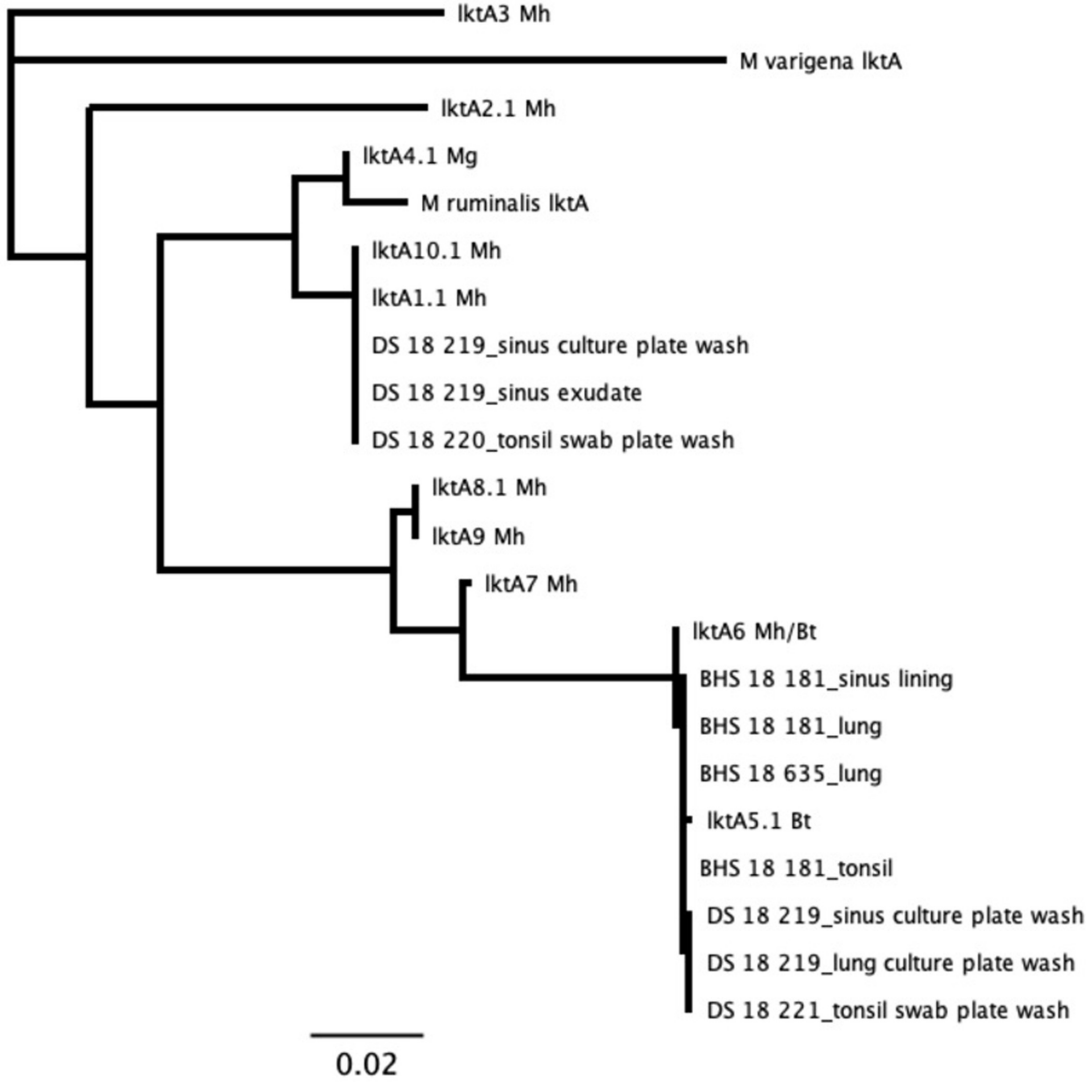
*Pasteurellaceae* leukotoxin A phylogenetic tree for samples from in-contact bighorn and domestic sheep. Sequences were identified in eight of 17 samples for which *M*. *haemolytica* or *B*. *trehalosi* was detected by MLST analysis. One sample (DS 18 219 sinus culture plate wash) produced a *B*. *trehalosi lktA* sequence but no *B*. *trehalosi* MLST sequence (see Figs 2 and 3). Although all of the *B*. *trehalosi lktA* sequences clustered with the lktA5.1 allele, there is minor separation between the bighorn and domestic sequences. Tree produced with neighbor-joining algorithm and no outgroup. DS = domestic sheep; BHS = bighorn sheep.

For samples that were cultured, results from our assay were in agreement with culture results (S15 Table). *Mannheimia* species were only detected from domestic sheep samples. All *Mannheimia* sequences, representing tissues, exudates, and cultures from two domestic sheep, tightly clustered as a single strain with the *M*. *haemolytica* genotype 2 [23] reference sequence (Fig 5). We identified sequences for *B*. *trehalosi* within samples from all three bighorn sheep and two domestic sheep. The bighorn sheep sequences clustered together as a unique strain, while domestic sheep sequences formed two distinct strains. There was no evidence of shared strains of *B*. *trehalosi* between the domestic and bighorn sheep (Fig 6). Sequences for *P*. *multocida* were identified in the bighorn ram that was found dead and the domestic ram that was apparently healthy but found to have chronic upper respiratory disease at necropsy. The sequences represented two unique strains, one from each animal. There was no evidence of shared strains of *P*. *multocida* between the domestic and bighorn sheep (Fig 7). Sequences for *M*. *ovipneumoniae* were identified in one bighorn sheep and two domestic sheep. The sequences represented two unique strains, one from the bighorn sheep and one from the two domestic sheep. There was no evidence of shared strains of *M*. *ovipneumoniae* between the domestic and bighorn sheep (Fig 8). For the *Pasteurellaceae lkt*A analysis, of 17 *M*. *haemolytica* or *B*. *trehalosi* sequences identified by MLST, only eight had *lktA* sequences identified. One sample produced a *B*. *trehalosi lktA* sequence but not a *B*. *trehalosi* MLST sequence. Within the *B*. *trehalosi lkt*A sequences, although bighorn and domestic sheep sequences were closely clustered, there was minor separation between the two groups (Fig 9).

Based on 16S rRNA results, the most significant shared organisms between bighorn and domestic samples were those targeted by the MLST analysis. One additional shared organism of possible significance was genus *Trueperella*, often associated with suppurative processes in wild and domestic ruminants (S1 File). Quantitative 16S rRNA classification was most relevant to libraries created from tissue samples, as libraries from cultures reflected selective growth of bacteria. Several trends are apparent in the tissue library results including the predominance of anaerobic bacteria identified (S5 Fig), and predominance of genus *Pasteurella* (likely representing *Pasteurella multocida*) among aerobic bacteria in sinus lining from the deceased bighorn and sinus exudates from the culled domestic sheep (S6 Fig).

## Discussion

We developed a culture-independent assay to assess bacterial populations from bighorn and domestic sheep respiratory samples, using extracted DNA from tissues and cultures. Our MLST analysis accurately identified type strain bacteria, and was a useful tool for examining relationships between targeted bacteria in domestic and bighorn sheep tissue and culture samples. The 16S rRNA analysis provided a nonbiased approach to examine tissue and exudate samples, as part of an effort to diagnostically recognize the polymicrobial nature of bighorn sheep respiratory disease. Limitations of the 16S rRNA analysis are expected to include variable performance of the 16S rRNA primers between bacteria, presence of MLST targets in samples that compete with 16S rRNA primers, and taxonomic biases in the NCBI 16S rRNA database. Despite these limitations, the 16S rRNA analysis adds diagnostic value to the MLST and *lkt*A analyses, providing context for interpretation and the opportunity for identification of nontarget bacteria.

This assay is not commercially available at the time of publication. However, the DNA extraction and multiplex PCR methods are easily conducted with basic molecular diagnostics equipment. Although library preparation and DNA sequencing are more technical methods, commercially available services for library preparation and short-read sequencing are becoming more affordable and accessible as genomics technology is becoming a diagnostic standard. The bioinformatics for our assay require minimal labor due to the automated workflows provided here and posted on Github, and the Geneious platform was chosen for bioinformatics due to the user friendly interface for the novice user. Analysis parameters can be adapted to user needs, including adjustments to quality thresholds.

Although this project was conducted primarily for method development, we did have the opportunity to apply the method to a limited number of diagnostic samples. This clinical application involved a small number of animals and varying samples, and results should be interpreted in this context. Given these samples and limitations, we demonstrated shared bacterial strains within the bighorn herd (*B*. *trehalosi*) and within the domestic herd (*M*. *haemolytica*, *B*. *trehalosi*), but we found no evidence of shared pathogen strains between the bighorn sheep and domestic sheep herds. This does not rule out shared pathogens, and possible limitations of our samples include limited sampling of the two domestic ewes in the study that had no post-mortem tissues evaluated, and postmortem interval for the bighorn sheep tissues evaluated. We did identify highly similar *B*. *trehalosi lkt*A genes within bighorn and domestic sheep samples. This finding could be of interest in the context of a larger sample size but we did not have sufficient data to make further interpretations for this case study.

Although the timing of the bighorn sheep death was concerning and generated a reasonable hypothesis regarding pathogen transmission from domestic sheep, it is important to have tools to test such hypotheses. Given the long history of respiratory disease and poor performance in the bighorn sheep herd, it is also reasonable to hypothesize that endemic respiratory pathogens in the bighorn herd were responsible for the mortality. However, the bighorn that died from pneumonia did have evidence of infection with at least two bacteria that were not detected in his healthy herd mates and therefore may have been recently acquired (*M*. *ovipneumoniae* and *P*. *multocida*). The pneumonic bighorn ram from this study did conduct a 34 km (one-way) foray over an 11 day period, 45 days prior to death, outside of bighorn sheep range but in an area known to hold other domestic sheep. Unfortunately, no observations were made by agency officials, nor were reports of the public made of the ram while conducting this long distance movement. No testing was done on domestic sheep in that area. It is also important to remember that bighorn sheep respiratory disease is a multifactorial problem and other infectious (viral, lungworm, others) and noninfectious (stress, habitat, others) variables can contribute to disease [10].

The assay described here has the potential to inform the management of bighorn sheep respiratory disease. MLST data can identify introductions of new pathogen strains and create pathogen profiles in herds to track changes in flora over time. Quantitative 16S rRNA data can provide context for MLST results, and identify trends in samples, such as those observed between diseased and healthy animals in this study. For example, our results suggest that *P*. *multocida* can predominate the respiratory flora in cases of chronic upper respiratory disease. This type of information may assist with identifying targets for test and cull efforts ongoing in herds with chronic respiratory disease and poor lamb recruitment [34].

## Supporting information

S1 Table Primer sequences used for multilocus sequencing typing, *lktA* identification, and 16S rRNA assessment in of bighorn sheep respiratory samples. (PDF)

S2 Table Parameters used for trimming merged, paired reads following Illumina sequencing. (PDF)

S3 Table Parameters used to assemble library reads to reference sequences. (PDF)

S4 Table Parameters used to re-assemble reads from filtered (to reference sequence) contigs to individual MLST loci. (PDF)

S5 Table Parameters used to generate consensus sequences for MLST loci. (PDF)

S6 Table Parameters used to produce assemblies for MLST concatenated sequences. (PDF)

S7 Table Matrix of distances for *lktA* alleles^a^. (PDF)

S8 Table Parameters used to produce contigs for *lktA* assembly. (PDF)

S9 Table Parameters for *lktA* assemblies, with results used for phylogenetics. (PDF)

S10 Table Parameters for error correction and normalization of reads prior to de novo assembly to identify 16S rRNA sequences. (PDF)

S11 Table Parameters used for de novo assembly of error corrected and normalized reads to identify 16S rRNA sequences. (PDF)

S12 Table Parameters used for classify sequences tool. (PDF)

S13 Table Parameters for maximum likelihood tree building for MLST phylogenetics. (PDF)

S14 Table Parameters for neighbor-joining tree for *lktA* phylogenetics. (PDF)

S15 Table Comparisons of MLST and 16S rRNA assay results with culture and conventional PCR results for tissues and swabs sampled from in-contact bighorn and domestic sheep. (PDF)

S1 Fig Validation of multilocus sequence typing assay showing alignments of reference sequences with MLST results for the corresponding reference bacteria libraries. Primers were not specific to *Mannheimia* species other than *M*. *haemolytica*, but nontarget *Mannheimia* species were accurately identified by the workflow. MLST loci are represented by green bars at bottom of alignments. Gray represents matches in the aligned sequences. Black bars represent mismatches, in these cases due to missing data.
(TIF)

S2 Fig Validation of multilocus sequence typing assay using mixed reads. Numbers in data labels represent proportion of reads from each of the two mixed libraries. MLST loci are represented by green bars at bottom of alignments. Gray represents matches in the aligned sequences. Black bars represent mismatches, in these cases due to missing data. Mh = *Mannheimia haemolytica*; Mr = *Mannheimia ruminalis*; Mg = *Mannheimia glucosida*; Bt = *Bibersteinia trehalosi*.
(TIF)

S3 Fig Alignments of expected *lktA* reference sequences with results from *lktA* workflow using libraries from type strains. Note that no *lktA* sequences were detected within the reads from the *M*. *varigena* type strain library, with missing data represented by black bar. This is likely due to primer mismatching in the expected reference sequence, as noted by yellow rectangles below mismatches in the primer (green triangles) sequences.
(TIF)

S4 Fig Coverage of 16S rRNA gene by reads from type strain bacteria libraries. Primers are marked by green triangle and represent (from left to right) V1 (start), V3 (start), V4 (end), and V9 (end).
(TIF)

S5 Fig Summary of 16S rRNA results for libraries from tissue samples from a deceased bighorn sheep with fatal respiratory disease (BHS 18 181) and an apparently healthy domestic sheep that was culled and had evidence of chronic upper respiratory disease at necropsy (DS 18 219). Results are provided at the highest taxonomic level classified, although classifications within these categories was as low as genus. Brown shaded segments represent anaerobic bacteria while blue shaded segments represent aerobic bacteria. Note the expansion of Proteobacteria and Actinobacteria in diseased tissue as compared to more healthy tissue. Proteobacteria primarily included *Pasteurella*, *Mannheimia*, and *Bibersteinia* genera while Actinobacteria primarily included genus *Trueperella*. DS = domestic sheep; BHS = bighorn sheep.
(TIF)

S6 Fig Summary of 16S rRNA results for libraries from sinus tissue from a deceased bighorn sheep with fatal respiratory disease (BHS 18 181), and sinus exudate from an apparently healthy domestic sheep that was culled and had evidence of chronic upper respiratory disease at necropsy (DS 18 219). Results are provided at the highest taxonomic level classified in panels to the left. Results are provided at the lowest taxonomic level classified in panels to the right. The right panels show results only for Proteobacteria. DS = domestic sheep; BHS = bighorn sheep.
(TIF)

S1 File Excel workbook of 16S rRNA analysis. (XLSX)
