# Supplementary material for: Assessing shared respiratory pathogens between domestic (Ovis aries) and bighorn (Ovis canadensis) sheep; methods for multiplex PCR, amplicon sequencing, and bioinformatics to characterize respiratory flora
Source: PLoS One. 2023 Oct 19;18(10):e0293062. doi: 10.1371/journal.pone.0293062 (PMC10586700; doi:10.1371/journal.pone.0293062)
Supplement: S1 Table — (PDF) [file pone.0293062.s001.pdf]

S1 Table. Primer sequences used for multilocus sequencing typing, *lktA* identification, and 16S rRNA assessment in of bighorn sheep respiratory samples.

Name	Sequence	Target (bp)	Conc (uM)	Reference
16S[a] 1492R	GGTTACCTTGTTACGACTT		0.3	Turner et al. 1999 (start V1)
16S 27F	AGAGTTTGATCMTGGCTCAG	1501	0.3	Lane 1991 (end V9)
16S 341F	CCTACGGGNGGCWGCAG		0.2	Herelemann et al. 2011 (V3-V4)
16S 805R	GACTACHVGGGTATCTAATCC	464	0.2	
Bt[b] adk fw	GTAAAGGCACGCAAGCACAA		0.2	Modified Pasteurella multocida and *Mannheimia haemolytica* pubMLST primers
Bt adk rv	CGATGATAAACAGTATTGCCCGC	550	0.2	
Bt atpD fw	CGCACTTGGTACATCAG		0.3	Modified from Christensen et al. 2004
Bt atpD rv	CAATCACTTCTTCAATAGAAC	1199	0.3	
Bt deoD fw	GCAGGTGCATTCGCTGATG		0.2	Modified *Pasteurella multocida* and *Mannheimia haemolytica* pubMLST primers
Bt deoD rv	AATTCCGCGGCAACACCATA	551	0.2	
Bt gapDH fw	TCGTATCGGTCGTATCGTAT		0.2	Modified *Mannheimia haemolytica* pubMLST primers
Bt gapDH rv	TTTTGCGTTGCTGTAGTTGC	519	0.2	
Bt infB fw	CTTGACTATATCCGTAAAGC		0.2	Modified from Christensen et al. 2004
Bt infB rv	GTTGCTACAGGACCACGACCTTTAT	530	0.2	
Bt mdh fw	GGGGAAGATCCTGCTTCAGC		0.2	Modified from *Pasteurella multocida* and *Mannheimia haemolytica* MLST primers
Bt mdh rv	ACCACTTCAGTACCTGCGTT	473	0.2	
Bt rpoB fw	GCAGTAAAAGAGTTCTTTGGTTC		0.2	Modified from Christensen et al. 2004
Bt rpoB rv	GTTGCATGTTCGCTCCCAT	562	0.2	
Bt zwf fw	TGATGAAGTCGCAAAAGTAC		0.2	Modified *Mannheimia haemolytica* MLST primers
Bt zwf rv	ACGGTTTACGCCATACTTTA	671	0.2	
lktA[c] fw	GCCCGTTATCTTGCGAATTT		0.3	Fox et al. 2015
lktA rv	TACCACCAAATAAGCGGTCA	946	0.3	
lktAext fw	TCAAGAAGARCTGGCAAC		0.3	Davies et al. 2001
lktAext rv	AGTGAGGGCAACTAAACC	3060	0.3	

Mh[d] adk fw	GCAAAGGTACGCAAGCTCAG		0.2	pubMLST
Mh adk rv	AAAATTTTCGCTAACTCAGCAC	569	0.2	
Mh aroE fw	GCTTCTGGAGCAAAAGGTTG		0.2	pubMLST
Mh aroE rv	CCTGTCCAACCAACATTCCT	580	0.2	
Mh deoD fw	TCCACACATTAACGCACCTG		0.2	pubMLST
Mh deoD rv	GCTCCATACTCTGCCGCTAC	576	0.2	
Mh gapDH fw	CCGTATCGGTCGTATCGTTT		0.2	pubMLST
Mh gapDH rv	TTTTGCGTTGCAGTAGTTGC	519	0.2	
Mh gnd fw	GTGATTGGACTCGCCGTAAT		0.2	pubMLST
Mh gnd rv	TTCGATACCGTTGTGAACCA	552	0.2	
Mh mdh fw	AGTAACCGGTTTTGCAGGTG		0.2	pubMLST
Mh mdh rv	GCTTTTGCCTCAACCACTTC	504	0.2	
Mh rpoB fw	GCAGTGAAAGAGTTCTTTGGTTC		0.2	Modified from *B. trehalosi* primers
Mh rpoB rv	GTTGCATGTTCGCACCCAT	559	0.2	
Mh zwf fw	TGATGAAGTCGCAAAAGTGC		0.2	pubMLST
Mh zwf rv	ACGGTTTTCGCCATACTTTG	671	0.2	
Mo[e] gyrB fw	GGGTCAAACAAAAGCAAAACTAAA		0.2	Cassirer et al. 2017
Mo gyrB rv	ACGGAATAAAAATGTCAAAAGTAA	547	0.2	
Mo gyrBext fw	AAAACGWCCAGGKATGTATATTGG		0.3	Cassirer et al. 2017
Mo gyrBext rv	GGATCCATTGTTGTTTCTCATAATTG	1701	0.3	
Mo IGS fw	GGAACACCTCCTTTCTACGG		0.2	Besser et al. 2012
Mo IGS rv	CCAAGGCATCCACCAAATAC	476	0.2	
Mo IGSext fw	GTTAACCTCGGAGACCATTG		0.2	Cassirer et al. 2017
Mo IGSext rv	GTTTGCTAGGTTGGGTTTCC	665	0.2	
Mo LMF	TGAACGGAATATGTTAGCTT		0.2	McAuliffe et al. 2003
Mo LMR	GACTTCATCCTGCACTCTGT	361	0.2	
Mo rpoB fw	TCGGCTTCAGCAATTCCTTTCTT		0.2	Cassirer et al. 2017
Mo rpoB rv	TCGGCTGTTGGGTTGTCTTCYC	680	0.2	

Mo rpoBE fw	AGTTATCACAATTTATGGATCAAA		0.3	Cassirer et al. 2017
Mo rpoBE rv	GCTCAAAGTTCCATTTCNCCGAA	1819	0.3	
Mo adk fw	TAATTGGCGCCCCTGGTTCA		0.2	Designed for this study
Mo adk rv	TTCGAAGCTTAACAATTTCAGGACG	489	0.2	
Mo atpA fw	TTGTTGGAGATGGAGTTGCG		0.2	Designed for this study
Mo atpA rv	GGCCAATTGCAACATAAACACA	501	0.2	
Mo gltX fw	ACTTTTAGGTTGAACTAGCGCTG		0.2	Designed for this study
Mo gltX rv	ATTCAGGACCTGATTCAGCTCA	500	0.2	
Mo tpiA fw	GTTATCGGGAATTGAAAAATGAATCA		0.2	Designed for this study
Mo tpiA rv	TGCTCAAATTGGTTCATAAGCTATT	501	0.2	
Pm[f] adk fw	AAGGBACWCAAGCVCAAT		0.2	pubMLST
Pm adk rv	CACTTTTTKYGTMCCGTC	569	0.2	
Pm deoD fw	GTGCATTTGCYGATGTTG		0.2	pubMLST
Pm deoD rv	TGSYGTKGTTTGTTCGTG	614	0.2	
Pm gdhA fw	YTTAGTTGARCCTGAACG		0.3	pubMLST
Pm gdhA rv	CTTGACCTTCAATYGTGC	1109	0.3	
Pm infB fw	CTTGACTACATTCGTAAAGC		0.2	pubMLST
Pm infB rv	GTTGCAACTGGACCACGACCTTTAT	482	0.2	
Pm mdh fw	AAGTTGCWGTWYTAGGTG		0.3	Modified from *B. trehalosi* primers
Pm mdh rv	CCTAATTCAATATCYGCACG	910	0.3	
Pm pgi fw	GCCWGTGYTKGTTGATGG		0.3	pubMLST
Pm pgi rv	TTGKGCTGGCGCRATRAA	907	0.3	
Pm rpoB fw	GCAGTGAAAGAATTCTTTGGTTC		0.2	Modified from *B. trehalosi* primers
Pm rpoB rv	GTTGCATGTTTGCACCCAT	520	0.2	

[a]16S = 16S rRNA gene target
[b]Bt = *Bibersteinia trehalosi* target
[c]lktA = Pasteurellaceae leukotoxin A gene target
[d]Mh = *Mannheimia haemolytica* target
[e]Mo = *Mycoplasma ovipneumoniae* target
[f]Pm = *Pasteurella multocida* target
