# Supplementary material for: Assessing shared respiratory pathogens between domestic (Ovis aries) and bighorn (Ovis canadensis) sheep; methods for multiplex PCR, amplicon sequencing, and bioinformatics to characterize respiratory flora
Source: PLoS One. 2023 Oct 19;18(10):e0293062. doi: 10.1371/journal.pone.0293062 (PMC10586700; doi:10.1371/journal.pone.0293062)
Supplement: S2 Table — (PDF) [file pone.0293062.s002.pdf]

S2 Table. Parameters used for trimming merged, paired reads following Illumina sequencing.

**Trimming Software**	BBDuk v 38.84
**Expose Options**	No
**Trim Adapters**	Yes
Adapters	All TruSeq, Nextera, and PhiX adapters
Trim	Right end
Kmer length	27
Maximum substitutions	1
Maximum substitutions + INDELs	0
Trim partial adapters from ends	No
**Trim Low Quality**	Yes
Trim	Both ends
Minimum quality	20
**Trim Adapters Based on Paired Read overhangs**	Yes
Minimum overlap	20
**Discard Short Reads**	Yes
Minimum length	200 bp
**Trim Low Complexity (Entropy)**	No
**Keep Original Order**	Yes
**Custom BBDuk Options**	None
