# Supplementary material for: Assessing shared respiratory pathogens between domestic (Ovis aries) and bighorn (Ovis canadensis) sheep; methods for multiplex PCR, amplicon sequencing, and bioinformatics to characterize respiratory flora
Source: PLoS One. 2023 Oct 19;18(10):e0293062. doi: 10.1371/journal.pone.0293062 (PMC10586700; doi:10.1371/journal.pone.0293062)
Supplement: S3 Table — (PDF) [file pone.0293062.s003.pdf]

S3 Table. Parameters used to assemble library reads to reference sequences.

**Mapping Software**	Bowtie2 v 7.2.1
**Expose Options**	No
**Data**	
Dissolve contigs and reassemble	Yes
Reference sequence	7 sequences (7 type strain reference seqs)
Assemble by name	No
Assemble each sequence list separately	No, use “For Each Document” in workflow
**Method**	
Mapper	Bowtie2
Alignment type	End to end
Preset	No
**Trim Before Mapping**	Remove existing trim regions
**Results**	Save contigs
**Advanced**	
Pack reference sequence when building index	No
Additional command line parameters	None
Use multiple CPUs	Yes
Max mismatches (in seed)	1
Seed length	22
Min insert size	200
Max insert size	800
Report	Best match only
