# Supplementary material for: Assessing shared respiratory pathogens between domestic (Ovis aries) and bighorn (Ovis canadensis) sheep; methods for multiplex PCR, amplicon sequencing, and bioinformatics to characterize respiratory flora
Source: PLoS One. 2023 Oct 19;18(10):e0293062. doi: 10.1371/journal.pone.0293062 (PMC10586700; doi:10.1371/journal.pone.0293062)
Supplement: S4 Table — (PDF) [file pone.0293062.s004.pdf]

S4 Table. Parameters used to re-assemble reads from filtered (to reference sequence) contigs to individual MLST loci.

**Mapping Software**	Geneious v 2022.2.2
**Expose Options**	No
**Data**	
Dissolve contigs and reassemble	Yes
Reference sequence	8 sequences (8 MLST loci for reference seqs)
Assemble by name	No
Assemble each sequence list separately	No, use “For Each Document” in workflow
**Method**	
Mapper	Geneious
Sensitivity	Custom
Find structural variants, short insertions, & deletions	No
Find short insertions and large deletions	No
Fine Tuning	None (fast/read mapping)
**Trim Before Mapping**	Remove existing trim regions
**Results**	Save contigs
**Advanced**	
Minimum mapping quality	30
Trim paired read overhangs	Yes
Allow gaps	Yes
Maximum per read	1%
Maximum gap size	2
Minimum overlap	25
Minimum overlap Identity	90%
Word length	25
Index word length	15
Ignore words repeated >20 times	Yes
Maximum mismatches per read	10%
Accurately map reads with errors to repeat regions	No

Only map paired reads which	Map nearby
Maximum ambiguity	4
Search more thoroughly for poor matching reads	No
