# Supplementary material for: Assessing shared respiratory pathogens between domestic (Ovis aries) and bighorn (Ovis canadensis) sheep; methods for multiplex PCR, amplicon sequencing, and bioinformatics to characterize respiratory flora
Source: PLoS One. 2023 Oct 19;18(10):e0293062. doi: 10.1371/journal.pone.0293062 (PMC10586700; doi:10.1371/journal.pone.0293062)
Supplement: S5 Table — (PDF) [file pone.0293062.s005.pdf]

S5 Table. Parameters used to generate consensus sequences for MLST loci.

**Consensus Building Software**	Geneious v 2022.2.2
**Expose Options**	No
**Threshold**	Highest quality (60%)
Threshold for sequences without quality	65%
Assign quality	Total
If no coverage, call	?
If coverage <4, call	?
**Trim to Reference Sequence**	Yes
**Call Sanger heterozygotes**	>50%
