# Supplementary material for: Assessing shared respiratory pathogens between domestic (Ovis aries) and bighorn (Ovis canadensis) sheep; methods for multiplex PCR, amplicon sequencing, and bioinformatics to characterize respiratory flora
Source: PLoS One. 2023 Oct 19;18(10):e0293062. doi: 10.1371/journal.pone.0293062 (PMC10586700; doi:10.1371/journal.pone.0293062)
Supplement: S6 Table — (PDF) [file pone.0293062.s006.pdf]

S6 Table. Parameters used to produce assemblies for MLST concatenated sequences.

**Mapping Software**	Geneious v 2022.2.2
**Expose Options**	No
**Data**	
Dissolve contigs and reassemble	No
Reference sequence	Concatenated ref sequence (no primers)
Assemble by name	No
Assemble each sequence list separately	No, use "For Each Document" in workflow
**Method**	
Mapper	Geneious
Sensitivity	Medium Sensitivity/Fast
Find structural variants, short insertions, & deletions	No
Find short insertions and large deletions	No
Fine Tuning	None (fast/read mapping)
**Trim Before Mapping**	Remove existing trim regions
**Results**	Save consensus sequences
**Consensus Sequence Options**	
Threshold	Highest quality (60%)
Threshold for sequences without quality	65%
Assign quality	Total
If no coverage call	?
Trim to reference sequence	No
Call Sanger heterozygotes	>50%
**Advanced**	
Minimum mapping quality	30
Trim paired read overhangs	Yes
Minimum support for structural variant discovery	2 reads
Include insertions in structural variants	No
Map multiple best matches	Randomly

All other settings	Presets based on Sensitivity (above)
