# Supplementary material for: Assessing shared respiratory pathogens between domestic (Ovis aries) and bighorn (Ovis canadensis) sheep; methods for multiplex PCR, amplicon sequencing, and bioinformatics to characterize respiratory flora
Source: PLoS One. 2023 Oct 19;18(10):e0293062. doi: 10.1371/journal.pone.0293062 (PMC10586700; doi:10.1371/journal.pone.0293062)
Supplement: S7 Table — (PDF) [file pone.0293062.s007.pdf]

**S7 Table. Matrix of distances for Pasteurellaceae lktA alleles[a].**

| | lktA1.1 | lktA2.1 | lktA3 | lktA4.1 | lktA5.1 | lktA6 | lktA7 | lktA8.1 | lktA9 | lktA10.1 |
|---|---|---|---|---|---|---|---|---|---|---|
| lktA1.1 | | 92.384 | 85.982 | 98.013 | 88.19 | 88.411 | 92.163 | 93.267 | 93.267 | 100 |
| lktA2.1 | 92.384 | | 87.969 | 91.722 | 85.32 | 85.43 | 88.521 | 89.956 | 89.956 | 92.384 |
| lktA3 | 85.982 | 87.969 | | 86.203 | 83.885 | 84.106 | 85.32 | 85.762 | 85.762 | 85.982 |
| lktA4.1 | 98.013 | 91.722 | 86.203 | | 88.521 | 88.742 | 92.053 | 93.377 | 93.377 | 98.013 |
| lktA5.1 | 88.19 | 85.32 | 83.885 | 88.521 | | 99.779 | 96.026 | 94.592 | 94.592 | 88.19 |
| lktA6 | 88.411 | 85.43 | 84.106 | 88.742 | 99.779 | | 96.247 | 94.812 | 94.812 | 88.411 |
| lktA7 | 92.163 | 88.521 | 85.32 | 92.053 | 96.026 | 96.247 | | 98.565 | 98.565 | 92.163 |
| lktA8.1 | 93.267 | 89.956 | 85.762 | 93.377 | 94.592 | 94.812 | 98.565 | | 100 | 93.267 |
| lktA9 | 93.267 | 89.956 | 85.762 | 93.377 | 94.592 | 94.812 | 98.565 | 100 | | 93.267 |
| lktA10.1 | 100 | 92.384 | 85.982 | 98.013 | 88.19 | 88.411 | 92.163 | 93.267 | 93.267 | |

[a]Alleles as defined by Davies et al. (2001).
