# Supplementary material for: Assessing shared respiratory pathogens between domestic (Ovis aries) and bighorn (Ovis canadensis) sheep; methods for multiplex PCR, amplicon sequencing, and bioinformatics to characterize respiratory flora
Source: PLoS One. 2023 Oct 19;18(10):e0293062. doi: 10.1371/journal.pone.0293062 (PMC10586700; doi:10.1371/journal.pone.0293062)
Supplement: S9 Table — (PDF) [file pone.0293062.s009.pdf]

S9 Table. Parameters for *lktA* assemblies, with results used for phylogenetics.

**Mapping Software**	Geneious v 2022.2.2
**Expose Options**	No
**Data**	
Dissolve contigs and reassemble	Yes
Reference sequence	7 sequences (*lktA* reference sequences with primers trimmed)
Assemble by name	No
Assemble each sequence list separately	Yes
**Method**	
Mapper	Geneious
Sensitivity	Low Sensitivity/Fastest
Find structural variants, short insertions, & deletions	No
Find short insertions and large deletions	No
Fine Tuning	None (fast/read mapping)
**Trim Before Mapping**	Remove existing trim regions
**Results**	Save consensus sequences
**Consensus Sequence Options**	
Threshold	Highest quality (60%)
Threshold for sequences without quality	65%
Assign quality	Total
If no coverage call	?
Trim to reference sequence	No
Call Sanger heterozygotes	>50%
**Advanced**	
Trim paired read overhangs	Yes
Map multiple best matches	Randomly
All other settings	Presets based on Sensitivity (above)
