# Supplementary material for: Assessing shared respiratory pathogens between domestic (Ovis aries) and bighorn (Ovis canadensis) sheep; methods for multiplex PCR, amplicon sequencing, and bioinformatics to characterize respiratory flora
Source: PLoS One. 2023 Oct 19;18(10):e0293062. doi: 10.1371/journal.pone.0293062 (PMC10586700; doi:10.1371/journal.pone.0293062)
Supplement: S10 Table — (PDF) [file pone.0293062.s010.pdf]

S10 Table. Parameters for error correction and normalization of reads prior to de novo assembly to identify 16S rRNA sequences.

**Correction and Normalization Software**	BBNorm v 38.84
**Expose Options**	No
**Error Correction**	Yes
Sensitivity	Default settings
Mark	Uncorrectable errors by leaving nucleotide unchanged and assigning low quality
**Normalization**	Yes
Target coverage level	40
Minimum depth	6
**Custom BBNorm Options**	None
