# Supplementary material for: Assessing shared respiratory pathogens between domestic (Ovis aries) and bighorn (Ovis canadensis) sheep; methods for multiplex PCR, amplicon sequencing, and bioinformatics to characterize respiratory flora
Source: PLoS One. 2023 Oct 19;18(10):e0293062. doi: 10.1371/journal.pone.0293062 (PMC10586700; doi:10.1371/journal.pone.0293062)
Supplement: S11 Table — (PDF) [file pone.0293062.s011.pdf]

S11 Table. Parameters used for de novo assembly of error corrected and normalized reads to identify 16S rRNA sequences.

**Mapping Software**	Geneious v 2022.2.2
**Expose Options**	No
**Data**	
Dissolve contigs and reassemble	No
Assemble by name	No
Assemble each sequence list separately	No, use "For Each Document" in Workflow
**Method**	
Assembler	Geneious
Sensitivity	Low Sensitivity/Fastest
**Trim Before Assembly**	Remove existing trim regions
**Results**	Save consensus sequences
**Consensus Sequence Options**	
Generate new consensus from contig	Yes
Threshold	Highest quality (60%)
Threshold for sequences without quality	65%
Assign quality	Total
If no coverage call	?
Trim to reference sequence	No
Call Sanger heterozygotes	>50%
**Advanced**	
Don't merge variants with coverage over approximately	6
Merge homopolymer variants	Yes
Produce scaffolds	Yes
All other settings	Presets based on Sensitivity (above)
