# Supplementary material for: Assessing shared respiratory pathogens between domestic (Ovis aries) and bighorn (Ovis canadensis) sheep; methods for multiplex PCR, amplicon sequencing, and bioinformatics to characterize respiratory flora
Source: PLoS One. 2023 Oct 19;18(10):e0293062. doi: 10.1371/journal.pone.0293062 (PMC10586700; doi:10.1371/journal.pone.0293062)
Supplement: S12 Table — (PDF) [file pone.0293062.s012.pdf]

S12 Table. Parameters used for Classify Sequences Tool.

**Classify Sequences Software**	Classify Sequences tool, Genious v 2022.2.2
**Expose Options**	No
**Searching Database**	
Database folder	Predefined prior to running tool, location of local database as described in manuscript
Sensitivity	Medium Sensitivity/Fast
Minimum overlap	50 bp
Minimum overlap identity	90%
Index seed length	12 bp
Maximum gap size	10 bp
**Classification**	
Minimum overlap identify to classify	75%
Minimum % identity higher than next best result to classify	0.2%
Classify using taxonomy from	Database sequence taxonomy field
Taxonomic level separator	; (semicolon)
Minimum overlap identity to classify at lowest taxonomic level (genus)	95%
Minimum overlap identity to classify at second lowest taxonomic level (eg. family)	90%
Minimum overlap identity to classify at third lowest taxonomic level (eg. order)	85%
Treat gaps longer than a single base as a single mismatch	No
Use multiple loci	No
**Results**	No options selected, which generates a classification table and a summary table
