# Supplementary material for: Assessing shared respiratory pathogens between domestic (Ovis aries) and bighorn (Ovis canadensis) sheep; methods for multiplex PCR, amplicon sequencing, and bioinformatics to characterize respiratory flora
Source: PLoS One. 2023 Oct 19;18(10):e0293062. doi: 10.1371/journal.pone.0293062 (PMC10586700; doi:10.1371/journal.pone.0293062)
Supplement: S13 Table — (PDF) [file pone.0293062.s013.pdf]

S13 Table. Parameters for maximum likelihood tree building for MLST phylogenetics.

**Tree Building Software**	RAxML v 8.2.11
Nucleotide model	GTR GAMMA
Algorithm	Rapid bootstrapping and search for best-scoring maximum likelihood tree (-f a-x 1)
Bootstrap replicates	100
Parsimony random seed	1
Additional command line options	None
