# Supplementary material for: Assessing shared respiratory pathogens between domestic (Ovis aries) and bighorn (Ovis canadensis) sheep; methods for multiplex PCR, amplicon sequencing, and bioinformatics to characterize respiratory flora
Source: PLoS One. 2023 Oct 19;18(10):e0293062. doi: 10.1371/journal.pone.0293062 (PMC10586700; doi:10.1371/journal.pone.0293062)
Supplement: S14 Table — (PDF) [file pone.0293062.s014.pdf]

S14 Table. Parameters for neighbor-joining tree for *lktA* phylogenetics.

**Tree Building Software**	Geneious Tree Builder v 2022.2.2
Genetic distance model	Tamura-Nei
Tree build method	Neighbor-joining
Outgroup	No outgroup
Consensus tree options	None
