# Supplementary material for: Assessing shared respiratory pathogens between domestic (Ovis aries) and bighorn (Ovis canadensis) sheep; methods for multiplex PCR, amplicon sequencing, and bioinformatics to characterize respiratory flora
Source: PLoS One. 2023 Oct 19;18(10):e0293062. doi: 10.1371/journal.pone.0293062 (PMC10586700; doi:10.1371/journal.pone.0293062)
Supplement: S15 Table — (PDF) [file pone.0293062.s015.pdf]

S15 Table. Comparisons of MLST and 16S rRNA assay results with culture and conventional PCR results for tissues and swabs sampled from in-contact bighorn and domestic sheep.

**SampleID**	**Mannheimia colony ID**	**Mh[a] PCR (plate wash)**	**Mh MLST**	**16S rRNA (to genus)**
18 219_sinus exudate	nd[b]	nd	Detected	Detected
18 219_sinus lining	Not Detected	Detected	Detected (tissue, plate wash)	Detected (tissue, plate wash)
18 219_sinus culture M ovi broth	nd	nd	Detected	<0.00%
18_219_nasal swab	Detected	Detected	nd	nd
18 219_lung	Not Detected	Detected	Detected (tissue, plate wash)	Detected (tissue, plate wash)
18 219_tonsil	Detected	Not Detected	Not Detected	Detected
18 219_tonsil swab	Not Detected	Not Detected	nd	nd
18 220_tonsil swab	Detected	Detected	Detected	<0.00%
18 220_nasal swab	Not Detected	Not Detected	nd	nd
18 221_tonsil swab	Not Detected	Not Detected	Not Detected	Detected
18 221_nasal swab	Not Detected	Not Detected	Not Detected	Not Detected
18 221_nasal swab M ovi broth	nd	nd	Not Detected	Not Detected
**SampleID**	**Bibersteinia colony ID**	**Bt[c] PCR (plate wash)**	**Bt MLST**	**16S rRNA (to genus)**
18 219_sinus exudate	nd	nd	Not Detected	Detected
18 219_sinus lining	Not Detected	Detected	Detected (tissue)	Detected (tissue, plate wash)
18 219_sinus culture M ovi broth	nd	nd	Not Detected	<0.00%
18_219_nasal swab	Not Detected	Detected	nd	nd
18 219_lung	Detected	Detected	Detected (tissue, plate wash)	Detected (tissue, plate wash)
18 219_tonsil	Not Detected	Detected	Detected	Detected
18 219_tonsil swab	Detected	Detected	nd	nd
18 220_tonsil swab	Not Detected	Not Detected	Not Detected	Not Detected
18 220_nasal swab	Not Detected	Not Detected	nd	nd
18 221_tonsil swab	Not Detected	Detected	Detected	Detected
18 221_nasal swab	Not Detected	Not Detected	Not Detected	Not Detected
18 221_nasal swab M ovi broth	nd	nd	Not Detected	Not Detected

SampleID	Pasteurella colony ID	Pm[d] PCR (plate wash)	Pm MLST	16S rRNA (to genus)
18 219_sinus exudate	no culture	no culture	Detected	Detected
18 219_sinus lining	Detected	Detected	Detected (plate wash)	Detected (tissue, plate wash)
18 219_sinus culture M ovi broth	nd	nd	Not Detected	Detected
18_219_nasal swab	Not Detected	Detected	nd	nd
18 219_lung	Not Detected	Detected	Detected (plate wash)	Detected (tissue, plate wash)
18 219_tonsil	Not Detected	Detected	Not Detected	Detected
18 219_tonsil swab	Not Detected	Detected	nd	nd
18 220_tonsil swab	Not Detected	Not Detected	Not Detected	Not Detected
18 220_nasal swab	Not Detected	Not Detected	nd	nd
18 221_tonsil swab	Not Detected	Not Detected	Not Detected	Detected
18 221_nasal swab	Not Detected	Not Detected	Not Detected	Not Detected
18 221_nasal swab M ovi broth	nd	nd	Not Detected	Not Detected
**SampleID**	**Mycoplasma colony ID**	**Mo[e] PCR (M. ovi broth)**	**Mo MLST**	**16S rRNA (to genus)**
18 219_sinus exudate	nd	nd	Not Detected	Not Detected
18 219_sinus lining	Contaminated	Detected	Detected (M. ovi broth)	Detected (tissue, M. ovi broth)
18_219_nasal swab	Contaminated	Not Detected	nd	nd
18 219_lung	Contaminated	Not Detected	Not Detected	Not Detected
18 219_tonsil	nd	nd	Not Detected	Not Detected
18 219_tonsil swab	nd	nd	Not Detected	Not Detected
18 220_tonsil swab	nd	nd	Not Detected	Not Detected
18 220_nasal swab	Contaminated	Not Detected	nd	nd
18 221_tonsil swab	nd	nd	Not Detected	Not Detected
18 221_nasal swab	Contaminated	Detected	Detected (M. ovi broth)	Detected (M. ovi broth)

[a]Mh = *Mannheimia haemolytica*
[b]nd = not done
[c]Bt = *Bibersteinia trehalosi*
[d]Pm = *Pasteurella multocida*
[e]Mo = *Mycoplasma ovipneumoniae*

Culture and PCR Methods per Butler et al. 2018, S2Appendix
