# Supplementary figures and images for: Assessing shared respiratory pathogens between domestic (Ovis aries) and bighorn (Ovis canadensis) sheep; methods for multiplex PCR, amplicon sequencing, and bioinformatics to characterize respiratory flora
Source: PLoS One. 2023 Oct 19;18(10):e0293062. doi: 10.1371/journal.pone.0293062 (PMC10586700; doi:10.1371/journal.pone.0293062)

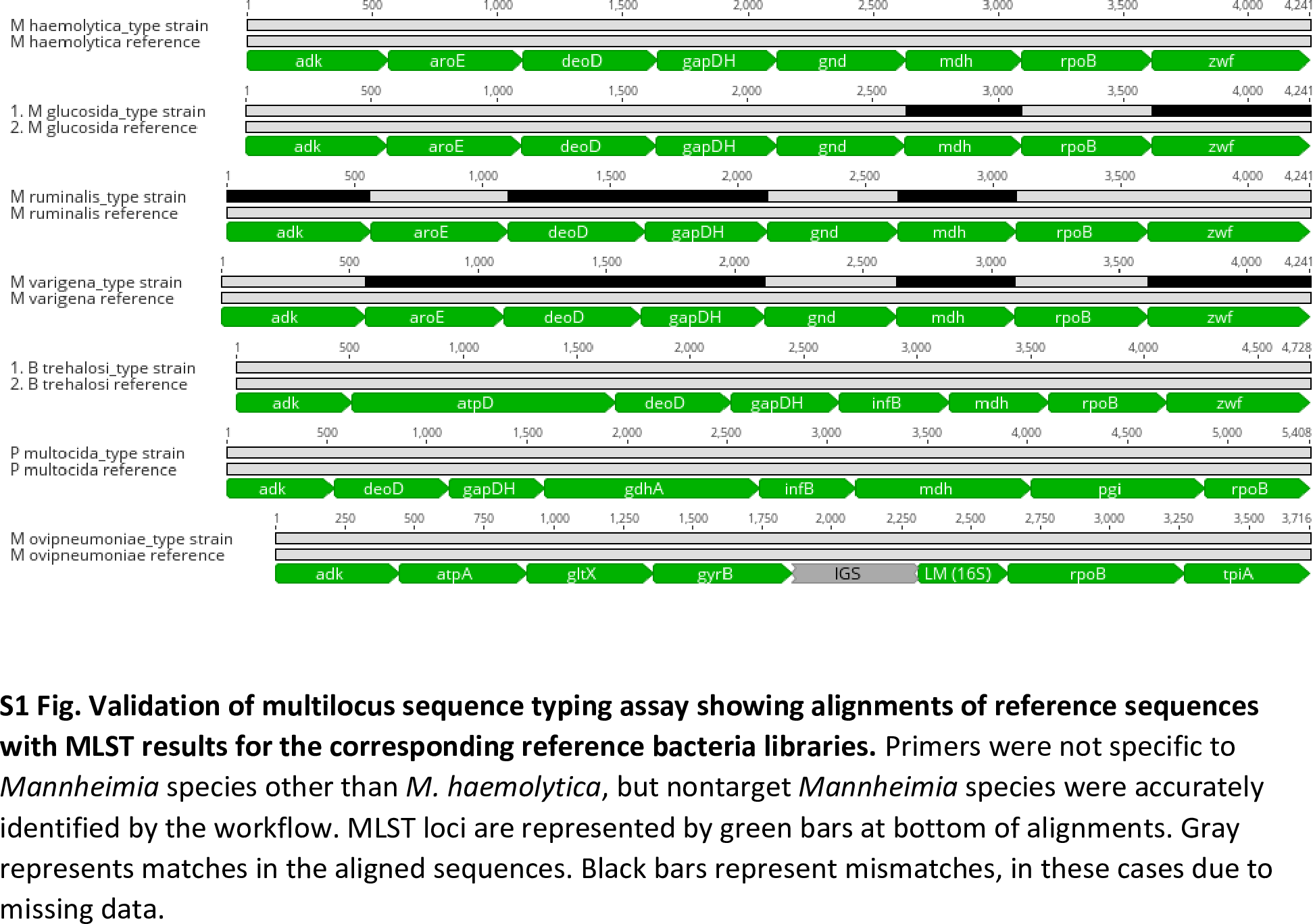

Supplement: S1 Fig — Primers were not specific to Mannheimia species other than M. haemolytica, but nontarget Mannheimia species were accurately identified by the workflow. MLST loci are represented by green bars at bottom of alignments. Gray represents matches in the aligned sequences. Black bars represent mismatches, in these cases due to missing data. (TIF) [file pone.0293062.s016.tif]

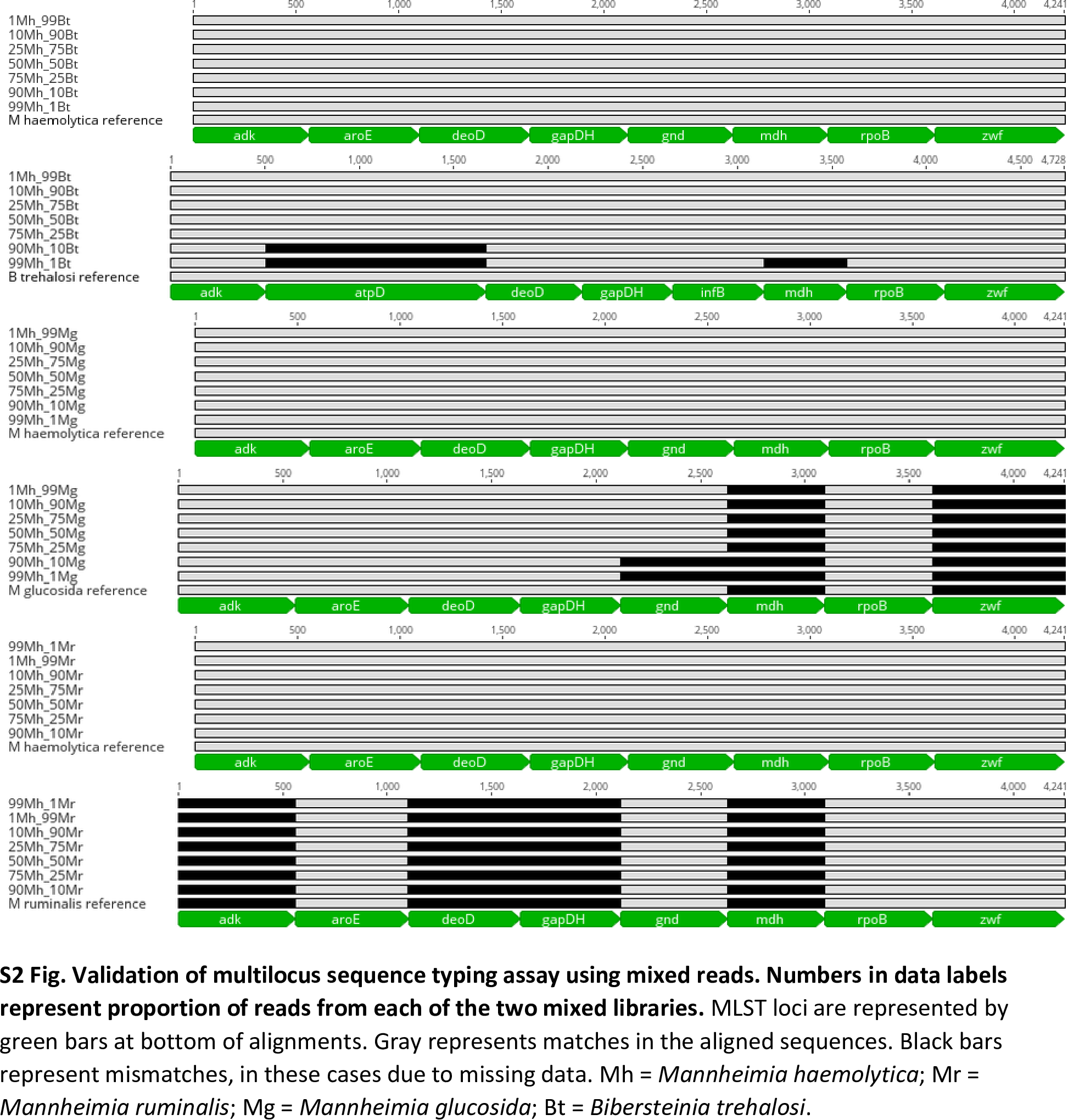

Supplement: S2 Fig — Numbers in data labels represent proportion of reads from each of the two mixed libraries. MLST loci are represented by green bars at bottom of alignments. Gray represents matches in the aligned sequences. Black bars represent mismatches, in these cases due to missing data. Mh = Mannheimia haemolytica; Mr = Mannheimia ruminalis; Mg = Mannheimia glucosida; Bt = Bibersteinia trehalosi. (TIF) [file pone.0293062.s017.tif]

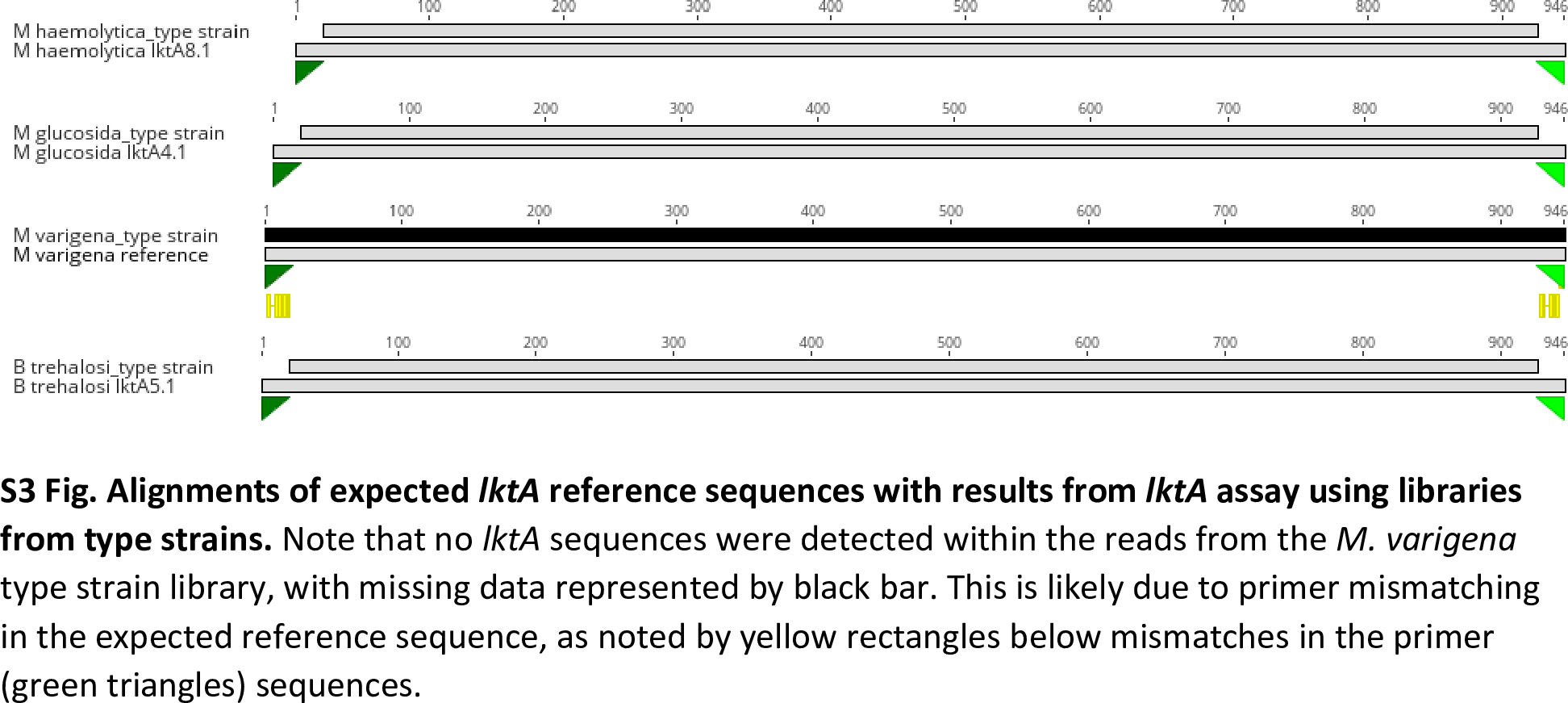

Supplement: S3 Fig — Note that no lktA sequences were detected within the reads from the M. varigena type strain library, with missing data represented by black bar. This is likely due to primer mismatching in the expected reference sequence, as noted by yellow rectangles below mismatches in the primer (green triangles) sequences. (TIF) [file pone.0293062.s018.tif]

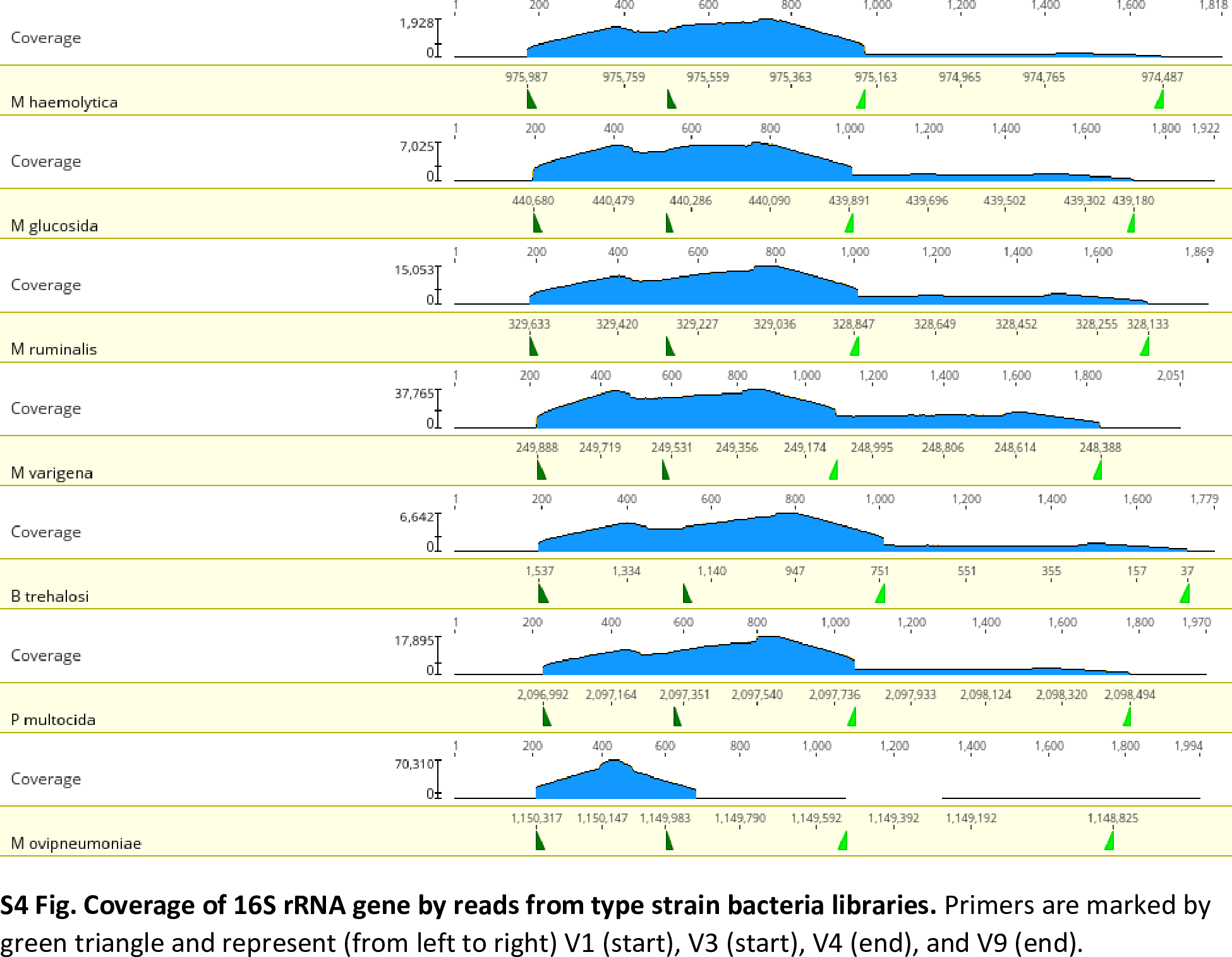

Supplement: S4 Fig — Primers are marked by green triangle and represent (from left to right) V1 (start), V3 (start), V4 (end), and V9 (end). (TIF) [file pone.0293062.s019.tif]

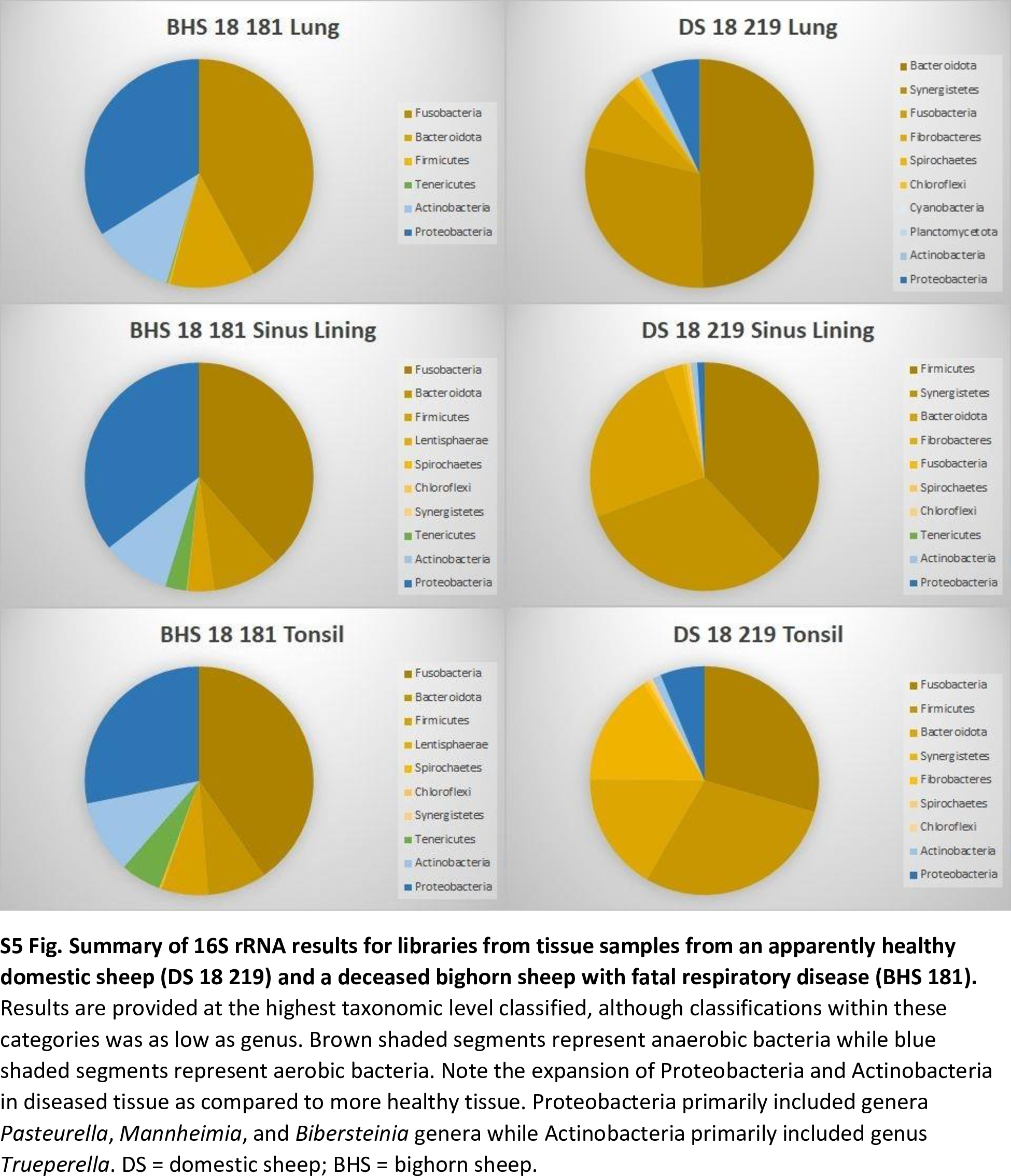

Supplement: S5 Fig — Results are provided at the highest taxonomic level classified, although classifications within these categories was as low as genus. Brown shaded segments represent anaerobic bacteria while blue shaded segments represent aerobic bacteria. Note the expansion of Proteobacteria and Actinobacteria in diseased tissue as compared to more healthy tissue. Proteobacteria primarily included Pasteurella, Mannheimia, and Bibersteinia genera while Actinobacteria primarily included genus Trueperella. DS = domestic sheep; BHS = bighorn sheep. (TIF) [file pone.0293062.s020.tif]

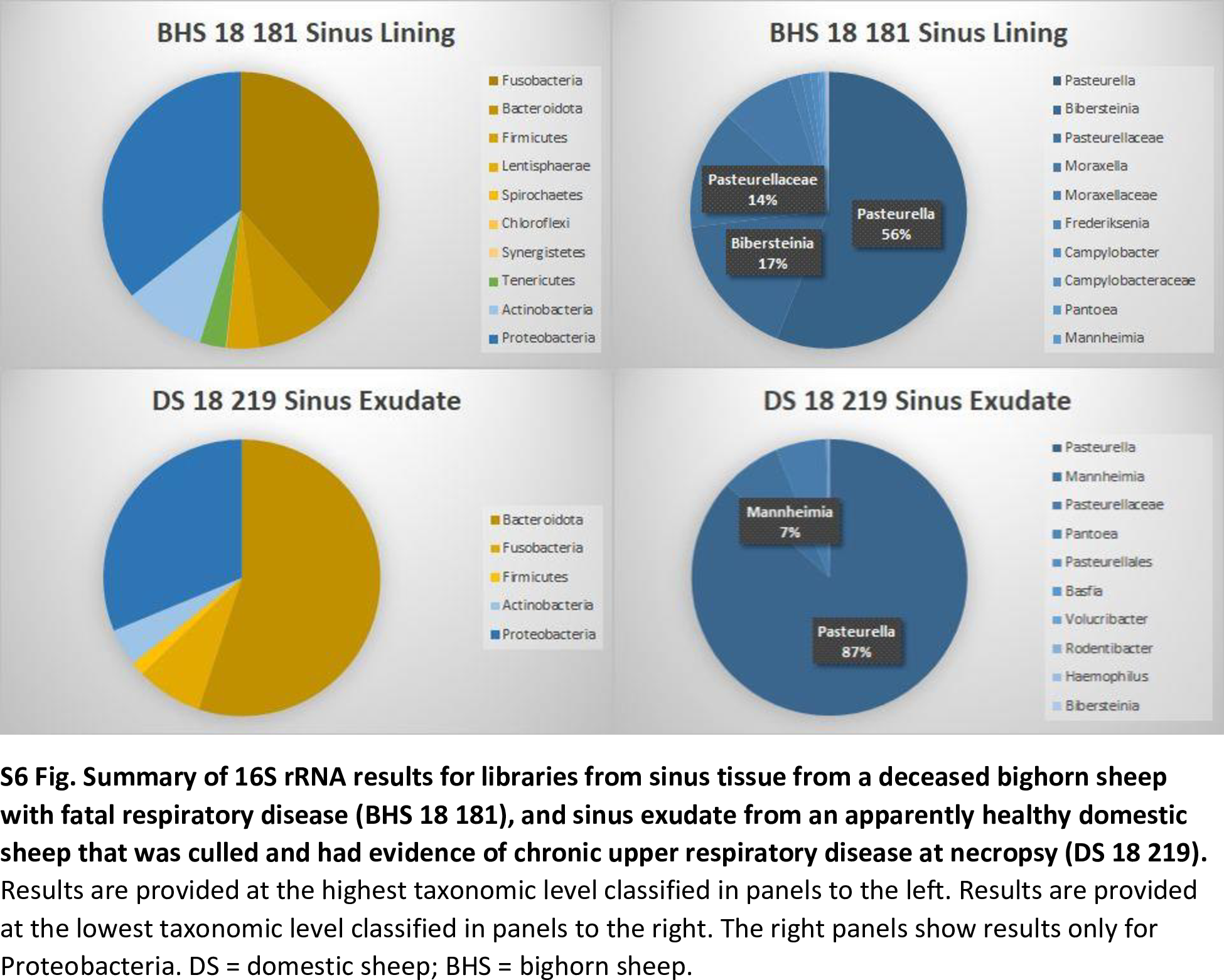

Supplement: S6 Fig — Results are provided at the highest taxonomic level classified in panels to the left. Results are provided at the lowest taxonomic level classified in panels to the right. The right panels show results only for Proteobacteria. DS = domestic sheep; BHS = bighorn sheep. (TIF) [file pone.0293062.s021.tif]
